# CRISPR Screens Identify Essential Cell Growth Mediators in BRAF Inhibitor-resistant Melanoma

**DOI:** 10.1016/j.gpb.2020.02.002

**Published:** 2020-05-13

**Authors:** Ziyi Li, Binbin Wang, Shengqing Gu, Peng Jiang, Avinash Sahu, Chen-Hao Chen, Tong Han, Sailing Shi, Xiaoqing Wang, Nicole Traugh, Hailing Liu, Yin Liu, Qiu Wu, Myles Brown, Tengfei Xiao, Genevieve M. Boland, X. Shirley Liu

**Affiliations:** 1 Clinical Translational Research Center, Shanghai Pulmonary Hospital, School of Life Sciences and Technology, Tongji University, Shanghai 200092, China; 2 Department of Medical Oncology, Dana-Farber Cancer Institute and Harvard Medical School, Boston, MA 02215, USA; 3 Department of Data Sciences, Dana-Farber Cancer Institute, Harvard T.H. Chan School of Public Health, Boston, MA 02115, USA; 4 Department of Clinical Laboratory, Shanghai Pulmonary Hospital, Tongji University School of Medicine, Shanghai 200433, China; 5 Center for Functional Cancer Epigenetics, Dana-Farber Cancer Institute, Boston, MA 02115, USA; 6 Center for Cancer Research, Massachusetts General Hospital, Harvard Medical School, Boston, MA 02114, USA; 7 Department of Surgery, Massachusetts General Hospital, Harvard Medical School, Boston, MA 02114, USA

**Keywords:** Drug resistance, CRISPR screen, Melanoma, BRAF inhibitor, Gene regulation

## Abstract

BRAF is a serine/threonine kinase that harbors activating mutations in ∼7% of human malignancies and ∼60% of melanomas. Despite initial clinical responses to BRAF inhibitors, patients frequently develop **drug resistance**. To identify candidate therapeutic targets for **BRAF inhibitor** resistant **melanoma**, we conduct CRISPR screens in melanoma cells harboring an activating *BRAF* mutation that had also acquired resistance to BRAF inhibitors. To investigate the mechanisms and pathways enabling resistance to BRAF inhibitors in melanomas, we integrate expression, ATAC-seq, and **CRISPR screen** data. We identify the JUN family transcription factors and the ETS family transcription factor ETV5 as key regulators of *CDK6*, which together enable resistance to BRAF inhibitors in melanoma cells. Our findings reveal genes contributing to resistance to a selective BRAF inhibitor PLX4720, providing new insights into **gene regulation** in BRAF inhibitor resistant melanoma cells.

## Introduction

Melanoma is an aggressive malignancy with a poor prognosis. Somatic mutations in *BRAF*, most commonly V600E or V600K [1], are the most frequent oncogene mutations in melanoma, and also appear recurrently in colorectal cancer, non-small cell lung carcinoma, and many other cancers [2]. *BRAF* encodes the serine/threonine protein kinase BRAF which belongs to the RAF family. This protein functions in regulating the MAPK/ERK signaling pathway, which affects the fundamental cellular processes such as differentiation, cell growth, and cell death [3]. The Ras–Raf–MEK–ERK pathway plays an essential role in tumor progression and metastasis as well [4].

The frequency of *BRAF* mutations in multiple cancer types and especially melanoma motivates the development of small molecules targeting mutant BRAF [3]. In early trials, patients with melanomas harboring activating *BRAF* V600E mutations show high levels of response to BRAF inhibitor (BRAFi) treatment, which makes it a promising therapeutic strategy [5], [6], [7]. BRAF inhibitors vemurafenib (PLX4032) and dabrafenib improve survival of *BRAF*-mutant melanoma patients compared to chemotherapy, which lead to FDA approval for this treatment in *BRAF*-mutant melanoma [8]. Although patients respond to BRAF inhibitors initially, the disease usually relapses with acquired resistance [9].

Numerous mechanisms of acquired BRAFi resistance have been reported. Amplification of the *BRAF* locus, *BRAF* alternative splicing, and secondary mutations in *BRAF* such as L514V and L505H confer resistance to BRAF inhibitors [6], [7], [10]. Hyper-activation of components in the RTK-Ras-ERK pathway [11], [12] and the persistent expression of the RTK platelet-derived growth factor receptor-β (PDGFRβ) or insulin growth factor-1 receptor (IGF-1R) [11], [13] can contribute to BRAFi resistance. Activation of other growth pathways, such as mTOR and PI3K, has also been associated with acquired resistance to BRAF inhibitors [14], [15]. Therefore, it is critical to comprehensively understand the mechanisms of resistance to BRAF inhibitors, and identify possible targets for combination therapies to counteract BRAFi resistance.

Most tumors, including melanoma, are considered a disease of abnormality in the cell cycle [16]. In melanoma, the *CCND1* amplification rate is 11%, and this increases to 17% in *BRAF* V600E melanoma, suggesting a critical role for *CCND1* in *BRAF*-mutated melanoma patients [17]. Elevated CDK4 activity also occurs in a subset of melanomas, and CDK4 has been implicated in BRAFi resistance [17]. Previous studies demonstrate that CDK4/6 inhibitors reduced melanoma cell growth and synergized with BRAF and MEK inhibitors [18], [19], [20]. These studies lead to clinical trials of combined inhibition of BRAF and CDKs. It is not known whether the efficacy of combined pan-CDK4/6 inhibitors with BRAF inhibitors is attributed to the inhibition of CDK4 or CDK6. Investigation into the mechanisms of BRAFi resistance will provide valuable knowledge about the gene regulation of melanoma tumorigenesis as well as how to avoid resistance and improve the efficacy of drugs.

To systematically investigate BRAFi resistance mechanism in melanoma, we conduct a series of experiments in *BRAF* (V600E)-mutated cell lines that had obtained resistance to the BRAFi PLX4032 following chronic exposure [11]. Specifically, our integrative analyses of CRISPR screens, transcriptome, and epigenetic profiling, reveal pathways and genes associated with BRAFi resistance and test candidate combination treatments to counteract BRAFi resistance.

## Results

### CRISPR knockout screens in melanoma cells with acquired resistance to BRAFi

To classify the genes whose loss of function may counteract resistance to BRAFi, we conducted CRISPR screens in a human melanoma cell line M238R1 [11]. The BRAFi-resistant cell line M238R1 was derived from long-term high-dose PLX4032 treatment of parental cell line M238 [11]. Although PLX4032 and PLX4720 are both BRAFi and structurally similar, a better response to PLX4720 is reported in the patient tumor-derived xenografts [21], [22]. To confirm the acquired resistance, we conducted a dose response assay with PLX4720 (Figure S1A). The IC_50_ value of the resistant line M238R1 was significantly higher than that of the parental line M238. Previous studies indicated that secondary mutations in *BRAF* could lead to BRAFi resistance [10]. To rule out the possibility that secondary mutations in *BRAF* lead to BRAFi resistance in M238R1, we sequenced the *BRAF* coding region in M238R1. We observed the V600E mutation as expected (Figure S1B), but no other secondary mutations in the *BRAF* coding region. Meanwhile, there is no *BRAF* amplification or alternative splicing variants that confer BRAFi resistance in this cell line [23]. This indicates that the drug resistance acquired by M238R1 is not due to a new genetic alteration inside the *BRAF* coding region.

**Figure 1:**
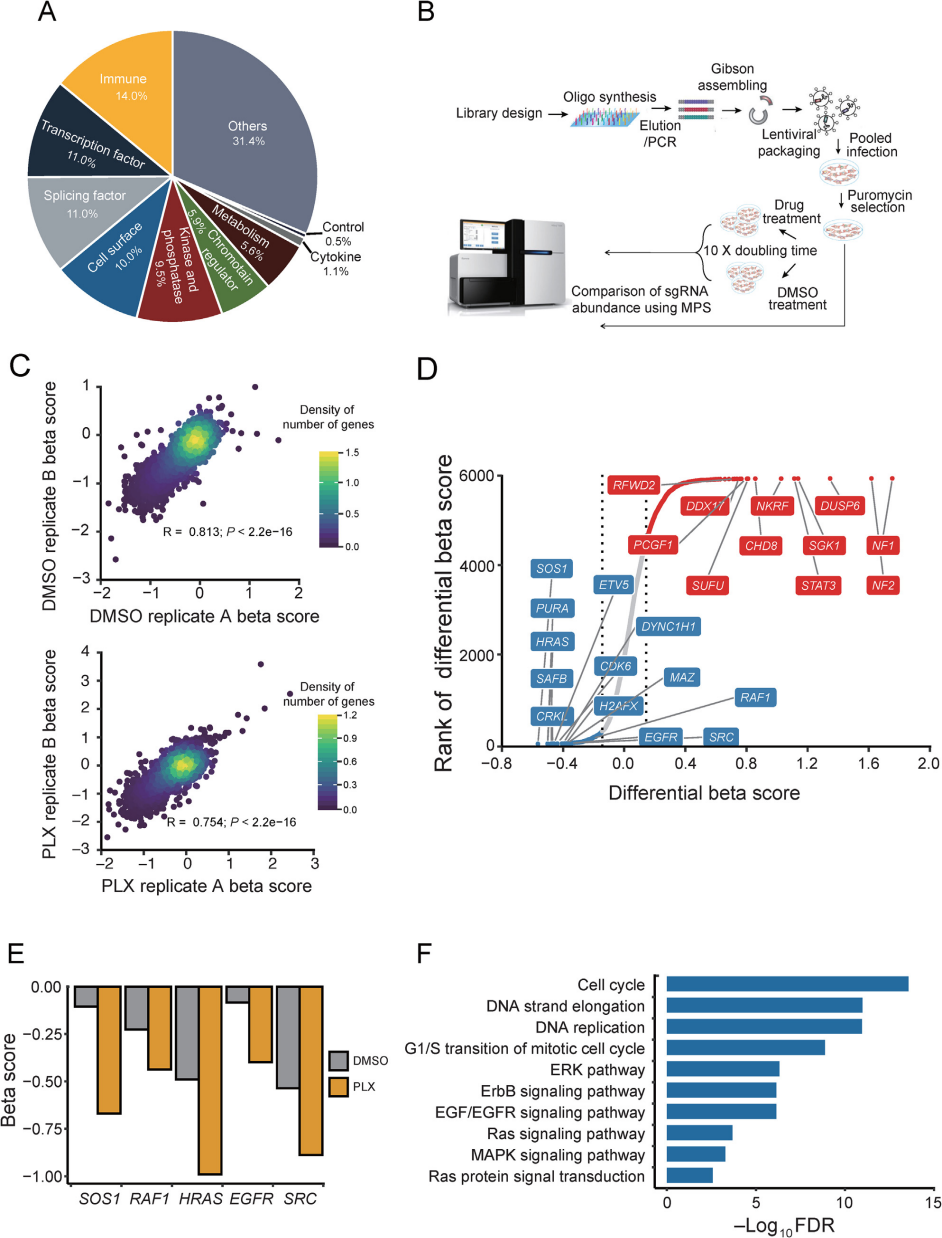
**Pooled CRISPR screens in a BRAFi-resistant melanoma cell line** **A.** Category of genes targeted by 6K-cancer sgRNA library. **B.** The workflow of CRISPR screens. **C.** Pearson correlation of beta score between two replicates (A, B) of M238R1 CRISPR screens. The treatments of DMSO (top panel) and PLX4720 (PLX) (bottom panel) were shown. **D.** Rank of the differential beta score between PLX treatment and DMSO PLX treatment. The two vertical lines indicated +/−1 standard deviation of the differential beta scores when comparing PLX treatment to DMSO treatment. Red dots indicate genes whose beta score increased upon PLX treatment, and blue dots indicate genes whose beta score decreased after PLX treatment. Genes whose beta score did not change significantly between different conditions are indicated with gray dots. **E.** Beta score of *SOS1*, *RAF1*, *HRAS*, *EGFR*, and *SRC* treated with PLX and DMSO, respectively. **F.** Pathway enrichment analysis of the 314 essential genes whose beta score decreased upon the PLX treatment compared to DMSO treatment. BRAFi, BRAF inhibitor.

To identify the genes that confer resistance to BRAF inhibition, we designed a new CRISPR sgRNA library targeting 6000 cancer-related genes (6K-cancer library, Table S1) based on COSMIC [24] and OncoPanel [25] (Figure 1A and Methods). We designed 19-bp sgRNAs against the gene coding regions using our predictive model [26]. For each gene, we selected 10 sgRNAs with optimized cutting efficiency and minimized off-target potential. The library contained 1466 sgRNAs against 147 genes essential for cell proliferation as positive controls [27], and 795 non-targeting sgRNAs along with 891 sgRNAs targeting *AAVS1*, *ROSA26*, and *CCR5* as negative controls. We performed two independent, pooled CRISPR screens by transducing a 6K-cancer library of lentivirus to M238R1 (Figure 1B). After viral transduction, we treated the melanoma cells with DMSO or 1 μM PLX4720, an optimal dose based on our preliminary tests (Figure S1A). After culturing for 14 days, we harvested cells from the different treatment groups and amplified the sgRNA sequences from the extracted genomic DNA. Then we quantified the abundance of sgRNAs through next-generation sequencing (NGS).

Screen data were analyzed by MAGeCK-VISPR, a comprehensive workflow for CRISPR screen data analyses [28]. MAGeCK-VISPR assesses the sgRNA abundance across different conditions and calculates a beta score for each gene under each condition compared to a designated control sample. A positive beta score, *i.e.*, the positive selection, indicates that silencing the corresponding gene provides a growth advantage under the experimental conditions. In contrast, a negative beta score, *i.e.*, the negative selection, indicates that silencing the gene confers a growth or survival disadvantage. Replicate screens from duplicate transductions exhibited a high correlation at the gene level (Figure 1C). To evaluate the quality of our CRSIPR screen, we checked the mapping ratio, the number of missing sgRNAs, the evenness of sgRNAs, and the distribution of the beta score maintained following the drug treatment (Figure S2). All of these results indicate that the screens functioned as designed.

Most positively selected or negatively selected genes behaved similarly under the control and treatment conditions (Table S2). Genes positively selected under both conditions were enriched for known tumor suppressors, such as *NF1* and *NF2*, as expected (Figure S3A and B). Consistent with previous work, genes identified as essential for the cell survival and proliferation under both growth conditions were significantly enriched in fundamental biological pathways, such as the ribosome, DNA replication, and RNA transport (Figure S3C and D). These results support a properly functioning CRISPR screen.

### Identification of essential genes for the growth of cells resistant to PLX4720

To explore genes that might play a role in the BRAFi resistance, we further analyzed the CRISPR screen data using MAGeCKFlute [27]. MAGeCKFlute facilitates comparison of beta score between different conditions. We robustly estimated σ, the standard deviation of the differential beta score by a “quantile matching” approach (Figure S4A). We identified genes whose beta score decreased with BRAFi treatment compared to DMSO treatment (Figure S4B and Table S2). Then, we selected 314 candidates whose depletion does not affect cell survival under DMSO mock treatment but become essential under the BRAFi treatment in M238R1. We ranked the identified hits by the change of the beta score (Figure 1D). Here, we labeled the top 10 genes whose beta score decreased with BRAFi treatment compared to DMSO treatment, such as *SOS1*, *PURA*, *HRAS*, *SAFB*, *CRKL*, *ETV5*, *CDK6*, *DYNCH1*, *H2AFX*, and *MAZ*. Among the 314 candidate genes, *SOS1*, *HRAS*, *SRC*, *EGFR*, and *RAF1* were previously reported to be involved in BRAFi resistance and were labeled in the rank plot as well [29], [30] (Figure 1D and E). Here, *SOS1* and *HRAS* are previously identified genes contribute to BRAFi resistance, which are also among the aforementioned top10 genes.

To further understand the pathways involved in the BRAFi resistance, we performed pathway analyses with the 314 candidate genes (Figure 1F). Among the network of genes whose beta score decreased after drug treatment, we found that the ErbB signaling pathway, Ras pathway, ERK pathway, MAPK pathway, and EGFR signaling pathway were highly enriched. These results are consistent with previous studies [11], [29], [31], [32]. Besides these known pathways, cell cycle and G1/S transition of mitotic cell cycle were the most enriched (Figure 1F). These newly discovered pathways were represented by *CDK6*, *CCND1*, *PSMB1*, and *RRM2* (Table S3).

### CDK6 confers resistance to BRAF inhibition in melanoma cells

**Figure 2:**
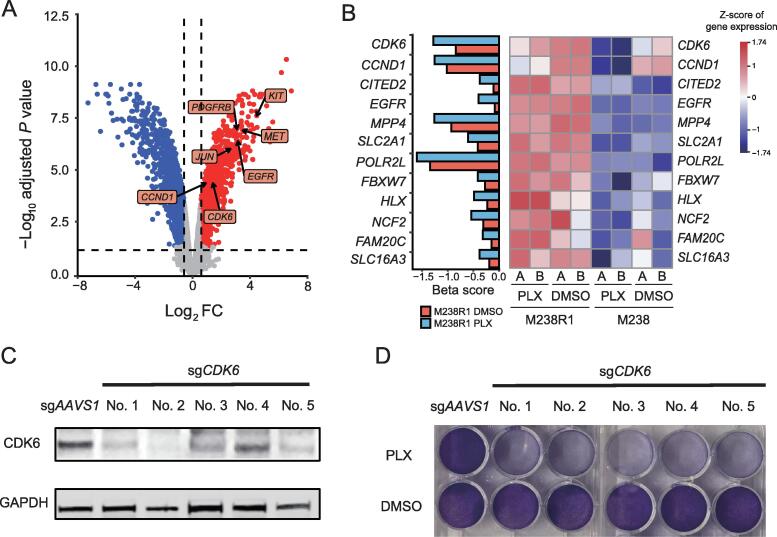
**Loss of *CDK6* sensitizes the M238R1 cell line to the BRAFi treatment** **A.** Volcano plot of differentially expressed genes between M238R1 and its parental cell line M238 under the treatment of PLX. The horizontal and vertical lines indicate the cutoff values (absolute FC ≥ 1.5; adjusted *P* ≤ 0.05). **B.** Beta score of the screens (left panel) and expression (right panel) of the intersect genes whose beta scores decreased under the PLX treatment condition and expression up-regulated in M238R1 cells. **C.** Western blotting analysis for the efficiency of *CDK6* sgRNAs. For gene knockout experiments, 5 independent CRISPR guides targeting *CDK6* were used, with one CRISPR guide targeting *AAVS1* for control. GAPDH is the loading control **D.** Colony formation assay of *CDK6* depletion under PLX treatment. Shown are the results from one representative experiment of two replicates.

We next sought to determine whether any genes related to BRAFi resistance might be dysregulated in melanoma cells. To assess this, we analyzed previously generated gene expression profiles in M238 and M238R1 cells treated with PLX4032 or DMSO [11]. In M238 cells, PLX4032 induced widespread changes in gene expression (Figure S5A). Our pathway analysis of genes down-regulated upon PLX4720 treatment in M238 cells showed that the MAPK signaling pathway were enriched, consistent with previous studies [11], [12] (Figure S5B). M238R1 cells exhibited fewer differentially expressed genes upon BRAFi treatment (Figure S5C). We next analyzed the genes that were differentially expressed by comparison of M238R1 cells with M238 cells upon BRAFi treatment. Under BRAFi treatment, there were 1374 up-regulated and 1574 down-regulated genes in M238R1 cells relative to M238 cells (Figure 2A and Table S4). Our re-analyses confirmed the previously reported overexpression of *KIT*, *MET*, *EGFR*, and *PDGFRB* in M238R1 relative to M238 [11]. In addition, we found that expression of the cell cycle genes *CDK6*, and *CCND1*, as well as transcription factor (TF) gene *JUN*, was up-regulated in resistant cells compare to the parental cells (Figure 2A).

We next integrated the expression data and CRISPR screen data to identify the dysregulated genes related to BRAFi resistance. Within the 314 genes whose depletion sensitized cells to BRAFi, there were 12 genes, including *CDK6*, specifically over-expressed in BRAFi-resistant M238R1cells (Figure 2B). This suggests that these 12 genes might be associated with the resistance to BRAFi and mediate cell proliferation in the resistant line.

To explore the potential druggable targets for the BRAFi-resistant cells, we further filtered the candidates with DGIdb, a database of published information on druggable genes and drug–gene interactions [33]. DGIdb identified *CDK6* as a potential druggable target with the FDA approved drugs for BRAFi-resistant cells. CDK6 is a cyclin-dependent kinase regulated by cyclin D proteins in cell cycle. Altered expression of these cell cycle genes has been observed in multiple human cancers [34], [35]. The number of sgRNAs targeting *CDK6* were markedly reduced under the PLX4720 treatment compared to the DMSO treatment (Figure S6A), suggesting that loss-of-function of *CDK6* can cause cells to be sensitive to PLX4720. To validate this result from the initial screens, we used five independent sgRNAs to knockout *CDK6* in the M238R1 cell line (Figure 2C). Consistent with the screen results, *CDK6* depletion increased cell sensitivity to PLX4720 treatment in long-term colony-formation viability assays (Figure 2D). Most tumors, including melanoma, have an irregular G1-to-S transition, primarily due to dysregulation of CDKs activities [36], [37]. We wondered whether the increased essentiality we observed for CDK6 is a general feature of CDKs or is specific to CDK6. We specifically evaluated the changes in essentiality of other CDKs (Figure S6B). Among all CDKs, only *CDK6* was more highly expressed in the resistant cell line compared to the sensitive cell line (Figure 2A and B) and became more essential in the presence of BRAF inhibitor.

### Exploring the mechanism of gene regulation in BRAFi resistance through chromatin changes

Epigenetic alterations are important features of cancer cells and may play a crucial role in the development of drug resistance. To model the epigenetic features associated with BRAFi resistance, we used ATAC-Seq [38] to compare the chromatin accessibility difference between M238 and M238R1 cells treated with PLX4720. On average, we sequenced each sample at ∼50 million PE150 fragments and obtained ∼89% uniquely mapping ratio (Table S5). We evaluated the quality of deep-sequencing data using different parameters, such as the number of uniquely mapped reads, PCR bottleneck coefficient (PBC) score, number of high-quality peaks, fraction of non-mitochondrial reads in peak region (FRiP), and ratio of peaks overlapping with total DNaseI hypersensitive peaks (DHS) (Figure S7). The ATAC-seq profiles showed high-quality features according to the criteria defined by Cistrome DB with ChIP-Seq and chromatin accessibility data of human and mice [39].

**Figure 3:**
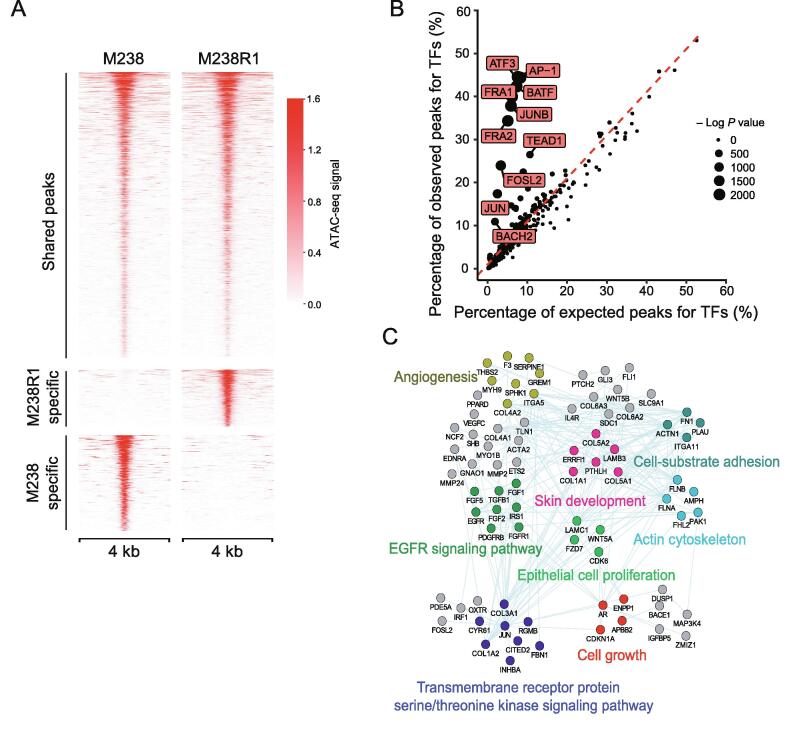
**DNA accessibility differs between BRAFi-sensitive and resistant cells** **A.** Genome-wide density plots of shared and specific ATAC-Seq peaks in M238 and M238R1 cell lines treated with PLX. Each row represents one peak. The color represents the intensity of chromatin accessibility. **B.** TF motif enrichment of M238R1-specific peaks. The percentage of expected (X axis) versus observed (Y axis) peaks for TFs was plotted. **C.** Network view of the genes whose expression was up-regulated in resistant cells treated with PLX and also associated with M238R1-sepecific peaks. Here, nodes represent proteins, edges connecting proteins represent possible interactions from the GeneMANIA database [65]. TF, transcription factor.

In total, 113,725 peaks were called in M238 cells, and 96,038 peaks were identified in M238R1 cells. Of the distinct peaks, we identified the differentially accessible peaks in M238 cells (M238-specific peaks) and M238R1 cells (M238R1-specific peaks) (Figure 3A and Table S6). We aggregated the M238R1 specific peaks of accessible chromatin and estimated the enrichment of TF binding [40]. M238R1-specific peaks were enriched for genomic locations bound by the AP-1 superfamily, including ATF3, JUNB, AP-1, BATF, and JUN (Figure 3B). To investigate the relationship between activated TFs and their target genes, we integrated the ATAC-seq data with gene expression data. We identified the genes that showed up-regulated expression in M238R1 treated with BRAFi and were also associated with M238R1-specific peaks. These genes were related to EGFR signaling, epithelial cell proliferation, skin development, and angiogenesis (Figure 3C), which are fundamental biological processes of melanoma development. Therefore, analysis of the ATAC-seq data and the expression data revealed a set of TFs and their target genes that were associated with BRAFi resistance.

### Identification of the JUN family and ETV5 as key regulators of ***CDK6***

**Figure 4:**
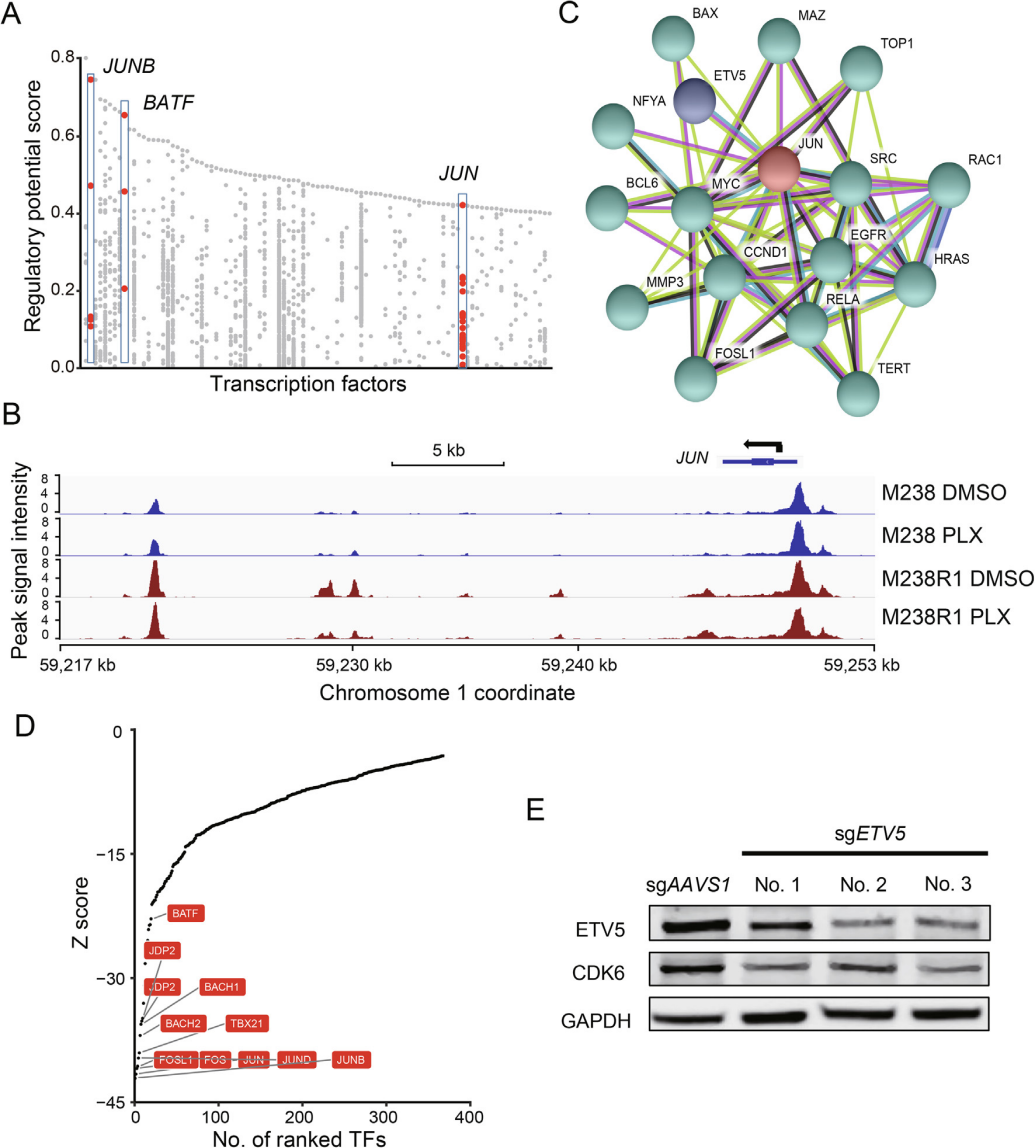
**Deficiency of *CDK6* or *ETV5* combined with PLX inhibits cell proliferation of BRAFi-resistant cells** **A.** TFs with the potential to regulate *CDK6* expression. The Y axis represents the regulatory potential score which were calculated by Cistrome DB Toolkit [39]. The X axis represents the different TFs. Each dot represents one ChIP-seq sample. **B.** Browser representation of the region near *JUN* from ATAC-seq of M238 and M238R1 under the different treatments. **C.** Interaction of TF JUN and genes whose essentiality increased after PLX treatment. Interacting partners of TF JUN were predicted using STRING database. JUN and ETV5 were individually labeled by the different colors to distinguish from the other proteins. Colored lines indicate different sources of evidence for each interaction. **D.** Rank plot of the TFs whose motif was enriched in the ETV5 ChIP-seq peaks. The Z scores were calculated according to their sequence logo similarity using MDSeqPos, available in Cistrome. For the negative “Z score”, the smaller ones mean significantly enriched. **E.** Validation of *ETV5* knockout in M238R1 cells by Western blotting using indicated antibodies. For gene knockout experiments, 3 independent CRISPR guides targeting *ETV5* were used, with one CRISPR guide targeting *AAVS1* for control. GAPDH is the loading control for Western blotting.

To identify the TFs that regulate *CDK6* expression, we used the CistromeDB Toolkit [39]. The Toolkit allows users to find the factors that might regulate the user-defined genes through public ChIP-seq (protein factors and histone marks) and chromatin accessibility (DNase-seq and ATAC-seq) data. We found the AP-1 superfamily members JUN, JUNB, and BATF as the putative TFs regulating *CDK6* (Figure 4A), consistent with previous studies [41], [42]. While all of the TFs might regulate *CDK6*, both expression level (Figure 2A) and chromatin accessibility (Figure 4B) of *JUN* were higher in M238R1 cells. *JUN* acts as a key mediator of BRAFi resistance and its upregulation has been observed in clinically treated patient tumors upon BRAF inhibitor [43], [44]. *JUN* is also required for cell cycle progression [45]. As *CDK6* knockout restored sensitivity to BRAFi treatment in M238R1 cells (Figure 2C and D) and the expression of *CDK6* and *JUN* was up-regulated in M238R1 cells compare to the M238 cells (Figure 2A). We conclude that dysregulation of *CDK6* by JUN mediates resistance to BRAF inhibition in melanoma cells.

To assess other proteins that might act with JUN to regulate *CDK6*, we examined the set of proteins that physically interact with the JUN protein according to the STRING database and proteins encoded by genes whose essentiality increased after BRAFi treatment. We identified *ETV5* as being in both of these gene sets (Figure 4C). ETV5 is a TF of the ETS family, which controls cell cycle gene expression and contributes to tumorigenicity [46]. Increased expression of ETV TFs affects the sensitivity to MEK inhibition [47]. Motif enrichment analysis of ChIP-seq data enables the identification of TFs that may cooperate with ETV5. According to the Cistrome DB [39], the JUN binding motif was enriched from the peaks of ETV5 ChIP-seq data, suggesting that JUN family might be a co-factor of ETV5 (Figure 4D). Consistent with the hypothesis that ETV5, JUN, and JUNB directly regulate *CDK6*, these TFs have strong binding around the *CDK6* gene (Figure S8A). We found that *ETV5* deletion restored sensitivity to BRAF inhibition by PLX4720 in melanoma cells and *ETV5* was the top hit of the genes that were more essential in M238R1 cells under the BRAFi treatment (Figure 1D). Similar to *CDK6*, the normalized read counts of *ETV5* sgRNAs decreased under the DMSO treatment or PLX4720 treatment (Figure S8B and C). Finally, we experimentally validated that the depletion of *ETV5* decreases the expression of CDK6 (Figure 4E). These observations suggest that the up-regulation of CDK6 expression promotes the cell proliferation and contributes to BRAFi resistance in melanoma. And CDK6-mediated resistance to BRAF inhibition is collaboratively regulated by TFs JUN and ETV5.

### Dual inhibition of BRAF and CDK6 in BRAFi-resistant cell lines

**Figure 5:**
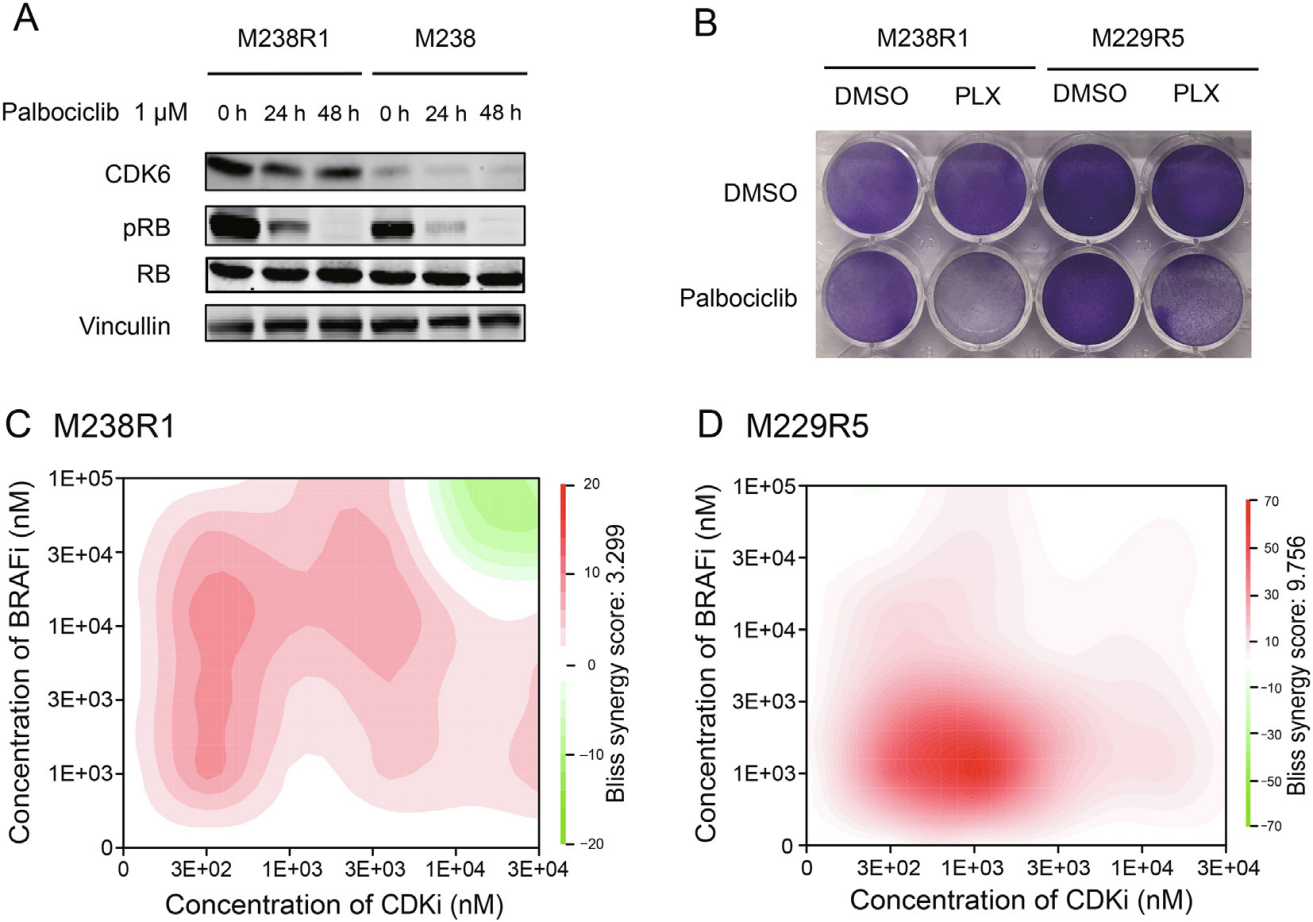
**Combination treatment of CDK6i and BRAFi overcomes BRAFi resistance *in vitro*** **A.** Immunoblot of lysates from M238 and M238R1 cells that were treated with 1 μM palbociclib for different time durations of 0–48 h. The blot is representative of two independent experiments. **B.** Colony formation assay for M238R1 and M229R5 under the combined treatment of palbociclib and PLX. **C.** The 2D drug synergy map of M238R1. **D.** The 2D drug synergy map of M229R5. The synergy score was calculated based on Bliss independence model.

Palbociclib is an inhibitor of CDK4 and CDK6 approved by the FDA for use in many cancer types [48]. CDK inhibitors or CDK4 depletion, combined with MEK inhibitors, were reported to effectively suppress cell growth in melanoma cells [19], [20]. However, whether the efficacy of a combination therapy of pan-CDK4/6 inhibitors with BRAF inhibitors is a general feature of CDK4/6 inhibition or is specific to inhibition of either CDK4 or CDK6 remains poorly understood. Here, we examined the changes in essentiality of the other CDKs (Figure S6B). Among all CDKs, only *CDK6* became more essential in the presence of BRAFi. Further we assessed the synergy between CDK6 and BRAF inhibition on BRAFi resistant cells. To verify the activity of palbociclib, we showed that 1 µM palbociclib effectively reduced the phosphorylation of RB1, a substrate of CDK6 (Figure 5A). We then treated M238R1 cells with CDK4/6 inhibitor palbociclib and/or PLX4720 and observed that inhibition of CDK6 sensitized BRAFi-resistant cells to PLX4720 treatment in a clonogenic assay (Figure 5B). To determine whether such combination treatment functions in other acquired drug-resistant cells, we also test the synergy of treatment combination in another cell line M229R5 [11]. M229R5 is derived from PLX4032-resistant sub-line M229 by chronic PLX4032 exposure and displays strong resistance to PLX4032 (Figure S9A and B). The combination treatment of palbociclib with PLX4720 was highly synergistic across a broad range of concentrations according to the Bliss independence model in the resistant lines (Figure 5C and D; Figure S10). These results suggest the potential of CDK6 and BRAF dual inhibition as a therapeutic strategy to overcome BRAFi resistance in our resistance model.

### ***CDK6*** expression is negatively associated with clinical outcome of ***BRAF***-mutant melanomas treated with BRAFi

**Figure 6:**
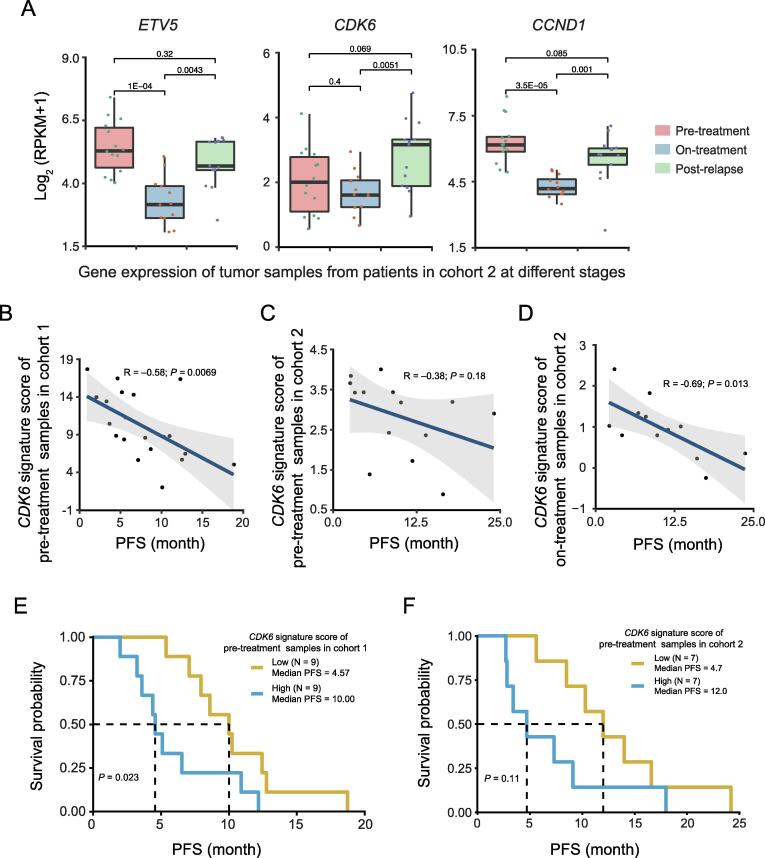
***CDK6* and *ETV5* expression is correlated with cancer progression in patients treated with BRAF inhibitors alone or together with MEK inhibitors** **A.** Expression of *ETV5*, *CDK6*, and *CCND1* in pre-treatment, on-treatment, and post-relapse samples of cohort 2 melanoma patients treated with BRAFi alone or BRAFi plus MEKi. Tumor samples for expression analysis were collected from patients before drug administration (pre-treatment), from patients without relapse during drug administration (on-treatment), and from patients with relapse during drug administration (post-relapse). Sampling details are provided in Table S7. There are 14 samples in the pre-treatment group, 12 samples in the on-treatment group, and 12 samples in the post-relapse group with RNA-seq data available. **B.** Correlation of PFS with the *CDK6* signature score of pre-treatment samples from patients in cohort 1. *CDK6* signature score was calculated based on the mean expression values of 10 genes from the *CDK6* signature panel. There are 18 samples in the pre-treatment group from the patients which are treated with BRAFi alone (13 patients) or BRAFi plus MEKi therapies (5 patients) afterwards. **C.** Correlation of PFS with the *CDK6* signature score of pre-treatment samples in cohort 2. There are 14 samples in the pre-treatment group from the patients which are treated with BRAFi alone (3 patients) or BRAFi plus MEKi therapies (11 patients) afterwards. **D.** Correlation of PFS with the *CDK6* signature score of samples in the on-treatment group from cohort 2. There are 12 on-treatment samples from the patients treated with BRAFi alone (3 patients) or BRAFi plus MEKi therapies (9 patients). **E.** Survival probability based on levels of *CDK6* signature in samples from the pre-treatment group of patients treated with BRAFi alone or BRAFi plus MEKi in cohort 1. **F.** Survival probability based on levels of *CDK6* signature in samples from the pre-treatment group of patients treated with BRAFi alone or BRAFi plus MEKi patients with melanoma cancer in cohort 2. PFS, progression free survival.

To investigate whether the expression of any validated BRAFi-resistant genes associated with BRAFi resistance in melanoma patients, we analyzed expression data from two independent cohorts treated with BRAF inhibitors (Table S7) [31], [49]. BRAF inhibitors used are vemurafenib or dabrafenib, whereas MEK inhibitors used are cobimetinib or trametinib. In cohort 1 [49], 18 patients were treated either with BRAFi alone (13 patients) or BRAFi plus MEKi therapies (5 patients). RNA-seq data on serial tumor biopsies of matched pre-treatment and post-relapse tumors were available (GEO: GSE65185). In cohort 2, 22 advanced melanoma patients were treated with BRAFi alone (7 patients) or BRAFi plus MEKi (15 patients) [31]. RNA-seq data of cohort 2 are not matched samples, but the pre-treatment, on-treatment, or post-relapse samples were available. The samples of cohort 2 were classified into 3 groups and RNA-seq data are available for 14 pre-treatment specimens, 12 on-treatment specimens, and 12 post-relapse specimens. Of the genes which were identified in our CRISPR screen (Figure 2B), *CDK6*, *CCND1*, and *ETV5* were more highly expressed in the tumors that have relapsed after BRAFi treatment relative to the on-treatment groups (Figure 6A).

We next examined whether up-regulated *CDK6* expression might be correlated with clinical resistance in some cases. To evaluate this, we created a 10-gene *CDK6* signature panel (*CDK6* signature), including *CDK6*, *AURKA*, *KIF23*, *TOP2A*, *BIRC5*, *MCM8*, *CDC25A*, *MKI67*, *CENPF*, and *PLK1*. This 10-gene proliferation signature completely overlapped the cell proliferation genes [31] and interacting partners of CDK6 predicted by STRING database. A negative correlation was observed between the expression of *CDK6* signature and the progression-free survival (PFS) in samples of both cohorts (Figure 6B–D). To further clarify the relationship between *CDK6* signature and clinical outcome not by the different drug treatment, we separated samples according to the different drug treatment conditions (BRAFi alone or BRAFi plus MEKi). High level of *CDK6* signature was correlated with poor PFS of melanoma patients treated with either BRAFi alone (Figure S11A) or BRAFi plus MEKi (Figure S11B and C). We used these ten genes to split the samples into low *CDK6* signature and high *CDK6* signature groups and assessed their prognostic value in melanoma patients of both clinical cohorts. Clinically, melanoma patients classified with high *CDK6* signature experienced shorter PFS than patients with low *CDK6* signature (Figure 6E and F). Consistent with this, high level of *CDK6* signature was associated with shorter PFS of the patients either treated with BRAFi alone (Figure S11D) or BRAFi plus MEKi (Figure S11E). These data suggest that high expression of genes functionally connected to CDK6 is associated with poor survival of BRAFi-treated melanoma patients. Overall, these observations suggest that *CDK6* up-regulated by TFs JUN and ETV5 might be associated with BRAFi resistance in melanoma patients.

## Discussion

Acquired resistance to therapies is frequent in clinical cancer treatment. BRAFi resistance is widely studied but remains a clinical challenge [11], [12], [14], [50]. For this reason, it is critical to investigate the mechanisms underlying drug resistance and design alternative therapeutic strategies to overcome drug resistance. Resistance to kinase inhibitors is often associated with secondary mutations in the target genes, which render the kinase insensitive to the inhibitor [10]. However, we did not find secondary mutations in *BRAF* coding regions in the M238R1. Reactivation of the MAPK pathway is another mechanism for the acquired resistance to BRAF inhibition [32]. But the levels of p-MEK1/2 and p-ERK1/2 decreased in M238R1 under BRAFi treatment [11]. Understanding how the cancer cells evade BRAF inhibition may promote the development of novel therapeutic strategies in *BRAF*-mutant melanoma patients and other *BRAF*-dependent tumors.

Several pooled CRISPR screens have been performed to identify mediators of drug resistance [51], [52]. In this study, we conducted CRISPR screens to systematically characterize resistance to BRAFi PLX4720 in melanoma. Our screen identified both previously known and novel genes related to BRAFi resistance. For instance, *CCND1*, *RAF1*, *EGFR*, and *SRC* were previously reported and identified by our screen as well [17], [29], [32]. Among the network of genes whose beta score decreased after drug treatment, we also found that the ErbB2 signaling pathway, regulation of Ras family activation, and EGFR signaling pathway represent examples of known pathway-dependent resistance mechanisms [11], [29], [31], [32], [53]. The cell cycle genes were enriched as a newly discovered class (Figure 1F), represented by *CDK6*, *CCND1*, *PSMB1*, and *RRM2*. These findings demonstrate the capacity of genome-wide CRISPR screens to reveal mechanisms of drug resistance.

Our approach also uncovered depletion of *CDK6* and *ETV5* restored the sensitivity to BRAF inhibition in BRAFi-resistant cells. To search for the key regulators of BRAFi resistance, we analyzed gene expression data, chromatin accessibility data, and our CRISPR screen data. Our observations indicate that overexpression of cell cycle gene *CDK6*, which is regulated by TFs JUN and ETV5, may confer resistance to BRAF inhibition. Indeed, a prior study suggested that overexpression of a single ETS TF confers resistance to MEKi trametinib in *KRAS* mutant pancreatic cancer, while suppression of *ETV1*, *ETV4*, or *ETV5* alone strongly decreased the resistance [47]. In a different previous study, researchers demonstrated that a high level of JUN was correlated with the inherent resistance to BRAFi/MEKi in melanoma cells [43]. However, JUN family members are not essential for the BRAFi-resistant cell lines. We hypothesize that many JUN family members could collaborate with ETV5 to regulate *CDK6*, such that the absence of any one member would not lead to cell death. Thus, our integrative analyses of the epigenetic and transcriptional data, together with genetic screening, provide insights into the regulation of BRAFi resistance in melanoma patients.

Palbociclib, an FDA approved drug established to target CDK4/6, has been evaluated in ∼30 different cancer indications [48], [54]. Combining palbociclib with PLX4720 reduced the proliferation of M238R1 and M229R5, which are both BRAFi-resistant melanoma cells. Indeed, prior studies implied that the combination of CDK4/6 inhibition and BRAFi halted the cell growth of several melanoma lines *in vitro* and *in vivo* [18], [19], [20]. However, these studies did not determine whether the efficacy of this combination was specific to the inhibition of CDK4 or CDK6. Here, we evaluated the essentiality of all CDKs in cells with acquired BRAFi resistance. Of all the CDKs, only *CDK6* was overexpressed in the resistant cells compared to the sensitive cells, and only *CDK6* became more essential in the presence of BRAFi (Figure S5B). Thus, our study demonstrates the feasibility of genome-wide pooled CRISPR-Cas9 knockout screens of resistant cells for uncovering genetic vulnerabilities that may be amenable to therapeutic targeting.

We found that *CDK6* knockout restored the drug sensitivity in the BRAFi-resistant cells and demonstrated that the CDK6 inhibitor palbociclib acts synergistically with BRAFi to halt cell growth in BRAFi-resistant cell lines. To further demonstrate the potential combination therapy, we tried to generate M238R1 xenografts. However, this effort failed, consistent with the reports from the lab that derived the resistant cell line (Lo Lab, personal communication). Additional evidence that *CDK6*, *ETV5*, and *JUN* may confer resistance to BRAF inhibition comes from our analysis of two independent melanoma cohorts. This analysis revealed high levels of *CDK6* and *ETV5* in tumors that acquire resistance to BRAFi treatment, thereby providing genetic evidence that these signaling pathways may be dysregulated upon BRAF inhibition. A high *CDK6* signature score was correlated with the worse PFS of melanoma patients in both clinical cohorts. These observations suggest that elevated global expression of *CDK6*, *JUN*, and *ETV5* modulates the response to BRAFi treatment. Our study strengthens this link by demonstrating that a combined inhibition of CDK6 and BRAF can overcome BRAFi resistance.

In conclusion, this study shows that there is a significant increase of CDK6 expression in the BRAFi-resistant cell lines and progressive tumors. Through loss-of-function screens, transcriptomics, and epigenetic profile analysis, we have identified a network that includes CDK6, ETV5, and JUN as the potential mechanism for BRAFi-resistant melanoma cells. Our findings offer new insights into resistance to BRAF inhibitors and support clinical studies of combined BRAF and CDK6 inhibition in a subset of activating *BRAF* mutations subject to relapse through acquired resistance.

## Materials and methods

### Cell culture and compounds

The paired human melanoma cell lines were gifts from the Roger Lo lab. The parental cell line M238 and M229 were established from patients' biopsies (UCLA IRB approval No. 02-08-067) [55]. And the BRAFi-resistant cell lines M238R1 and M229R5 were derived from long-term high-dose PLX4032 treatment of M238 [11]. Cells were sustained in Dulbecco’s modified Eagle medium (DMEM) including 10% fetal bovine serum (FBS), glutamine, and 1% penicillin/streptomycin. For packaging virus, HEK293T cells were grown in same medium with melanoma cell lines. All cell lines were mycoplasma free. Stocks of PLX4720 (catalog No. S1152) and palbociclib isethionate (PD0332991, catalog No. S1579) were purchased from Selleck Chemicals (Houston, TX).

### Library design

The customized library contains 6000 genes that were reported as cancer-related genes by multiple sources, including OncoPanel and Cosmic (Table S1). We designed multiple 19-nt sgRNAs, optimized cutting efficiency, and minimized off-target potentials with previously developed algorisms [26]. For each gene, we selected 10 best sgRNAs with high cutting efficiency score and low off-target potentials. The positive control and two types of negative control sgRNAs were incorporated into our library. The positive control contains 1466 sgRNAs targeting 147 core essential genes, which have been demonstrated as essential genes under multiple screen conditions. The first type of negative controls is the non-targeting sgRNAs, which contains 795 sgRNAs whose sequences are absent in the human genome. The second type of negative controls is 1891 sgRNAs targeting *AAVS1*, *ROSA26*, and *CCR5* that are considered as the safe-harbor regions.

### Cloning of individual sgRNAs and sgRNA libraries

For the 6K-cancer library, we used the lentiCRISPR v2 vector (plasmid No. 52961, Addgene, Watertown, MA) as backbone [56]. We designed ten sgRNAs per gene to target ∼6000 genes and added non-targeting sgRNAs as controls (Table S1). For library construction, we used the same protocol as previous CRIPSR screen [52]. For individual sgRNA cloning, we synthesized the pairs of oligonucleotides (IDT) containing the *Bsm*BI-compatible overhangs. We used the standard protocols to anneal and clone the sgRNA oligos into the lentiCRISPR v2 vector [56]. The sequences of individual sgRNAs for *CDK6* and *ETV5* are shown in Table S8.

### Virus production and infection

For each library to be transfected, we plated HEK293T cells in 25 ml of media in a 15-cm tissue culture plate. Typically, 20 μg vector DNA, 15 μg psPAX2 packaging plasmid, 6 μg pMD2.G envelope plasmid, and 200 μl transfection reagent (X-tremeGENE, Roche, Switzerland) were used; DNA and transfection reagent were separately pre-diluted in 3 ml serum-free OPTI-MEM and then mixed. After incubating for 15 min, the DNA and transfection reagent mixtures were added to HEK293T cells seeded in the dish. After 8 h–12 h, the media were changed to 25 ml fresh DMEM with 10% FBS and 1% BSA. Viral supernatant was collected from the medium two days after transfection. The viral supernatant was filtered through 0.45-μm membranes, and then infected the target cells with polybrene (8 μg/ml, Millipore, Burlington, MA). After 48 h of infection, puromycin (2 μg/ml) was used for selection over two days, which eliminated the uninfected cells.

### Pooled CRISPR screen

For the pooled CRISPR screen, a total of 1.2 × 10^8^ cells were infected with the pooled lentiviral library at a multiplicity of infection (MOI) of 0.3. After puromycin selection, the cells were divided into three groups (Day 0, DMSO, and PLX treatment). The cell pellet of Day 0 group was stored at −80 °C. For the two treatment groups, the cells were cultured for 14 days, treated with DMSO or 1 μM PLX4720 individually. The cells were cultured for 14 days and split every 2–3 days before genomic DNA extraction and library amplification.

### Amplification and sequencing of sgRNAs from cells

After harvesting the cell from different groups, we used QIAGEN (Germany) DNeasy Blood & Tissue Kit to extract genomic DNA according to the manufacturer’s instruction. Library construction for NGS were performed by PCR as previously described [56]. The PCR products were purified and then sequenced on a HiSeq 2500. Each library was sequenced at 30–40 million reads to achieve ∼300 × average coverage over the CRISPR library.

### CRISPR screen analysis

The CRISPR/Cas9 screening data were performed by MAGeCK and MAGeCK-VISPR algorithms [28]. MAGeCK-VISPR calculated the beta score for each gene. Comparison of the differential beta scores between the BRAFi treatment and DMSO treatment was performed using MAGeCKFlute [27], which was designed to perform quality control, normalization, and downstream analysis of the functional CRISPR screens.

### Microarray data analysis

The expression profiles of M238R1 and its parental cell line M238 were downloaded from the Gene Expression Omnibus database (GEO: GSE9340). *limma*, an R package, was used to perform differential expression analysis [57]. The absolute FC > 1.5 and Benjamini–Hochberg adjusted *P* < 0.05 were used as a cutoff to identify differentially expressed genes.

### ATAC-seq and data analysis

ATAC-seq library preparation was performed as the previously described Omni-ATAC protocol [58]. The concentration of the library was measured by Qubit 3.0 (Life Technologies, Rockville, MD), and the size distribution was evaluated by Agilent 4200 TapeStation system. ATAC-seq libraries were sequenced (35 bp paired-end) on the Illumina NextSeq 500. Quality control, reads alignment, and peak calling were performed by ChiLin [59]. BEDTools [60] ‘*merge*’ function was used to merge the M238 and M238R1 peaks. The ‘*coverage*’ function of BEDTools was used to create an input matrix used for detecting differentially accessible peaks. The DESeq2 R package was used to assess the differential peaks between different groups [61]. Peaks with a log_2_ FC > 1 and BH-adjust *P* < 0.05 were considered as differential peaks. The Genomic Regions Enrichment of Annotations Tool (GREAT) was used to annotate the M238R1-specific peaks. The identification of TF motifs that are enriched in M238R1-specific elements was performed using HOMER.

### ChIP-seq data mining in Cistrome DB

We used the Cistrome DB Toolkit function to investigate the TFs, which could regulate *CDK6* [39]. This function would return a list of the transcription factors that are most likely to regulate expression of *CDK6*. Regulatory potential (RP) scores calculated with the BETA algorithm [62] are from Cistrome DB. To identify the potential cooperative factors of ETV5, we used the analysis results from the Cistrome Data Browser [39]. High quality ETV5 ChIP-seq data (Cistrome Data Browser ID: 42714) were used to explore the potential cooperative factors of ETV5. The “QC Motifs” panel showed the significantly enriched motifs of other factors in the ETV5 ChIP-seq peaks.

### Western blotting analysis

For Western blotting, cells were lysed in RIPA buffer (Santa Cruz Biotechnology, Dallas, TX) with protease and phosphatase inhibitor cocktail (Cell Signaling Technology, Danvers, MA). Protein concentrations were measured with Thermo Fisher Scientific Bradford Assay (Catalog No. PI23236). ETV5 antibody (Catalog No. ab102010) was purchased from Abcam, and CDK6 antibody (Catalog No. sc-7961) was obtained from Santa Cruz Biotechnology. GAPDH antibody (Sigma, G9545, Saint Louis, MO), ERK2 antibody (Santa Cruz Biotechnology, sc-1647), and vinculin antibody (Santa Cruz Biotechnology, sc-73614) were used as a loading control. Both goat anti-rabbit and goat anti-mouse secondary antibodies were purchased from LI-COR Biosciences (Lincoln, NE). The fluorescent signals were captured with Odyssey CLX Imaging System (LI-COR Biosciences).

### Cell proliferation and colony formation assays

Responses to a single drug or combination treatment were evaluated by the CellTiter 96 cell proliferation assay from Promega. Cells were cultured in 96-well plates (2000 cells per well) for 18 h–24 h before treatment. The cells were treated with diverse concentrations of inhibitors for 3 days. The CellTiter 96 Solution were added into each well and incubate for 1 h–4h before the 490-nm absorbance reading on SpectraMax M2 (Molecular Devices, San Jose, CA). All experiments were performed in triplicate. For colony formation assays, 1000 cells were seeded in a 6-well plate, and attached for 24 h. The cells were maintained for two weeks after treated with different drugs. Colonies of cells were wash with PBS and then fixed with 1% methanol. The colonies were stained with 1% crystal violet and imaged.

### Drug synergy analysis

The SynergyFinder R package was used to assess the drug synergy [63]. The synergy scores were based on Bliss model.

### Patient sample collection and data analysis

We collected melanoma cancer datasets with BRAFi treatment, patient survival durations, and tumor gene expression profiles from NCBI. Finally, we used the expression data from two individual cohorts [31], [49]. The therapies for patients from the two cohorts are shown in Table S7. The expression data of cohort 1 (GEO: GSE65185) are available. The expression data of cohort 2 are provide by the Boland Lab. The expression values of all genes are expressed as RPKM. A Kaplan-Meier plot with log-rank test was employed to compare survival among groups.

## Authors’ contributions

XSL conceptualized the study and data analysis. ZL and BW conceived and designed the study. ZL performed all experiments including the screening and *in vitro* experiments. SG supervised experiments and provided technical support. BW performed computational analysis of the data. GMB and AS provided the data of the patients in cohort two. CHC designed the CRISPR screen library. TX constructed the CRISPR-sgRNA library. PJ, TH, QW, and SS participated in cell culturing. PJ, HL, YL, XW, NT, and MB contributed to the discussion. XSL, ZL, and BW wrote this manuscript with feedback from the other authors. The final manuscript has been read and approved by all authors.

## Competing interests

TX and XSL are founders of GV20 Oncotherapy.

## Data Availability

The ATAC-seq data have been deposited in the Genome Sequence Archive [64] at the National Genomics Data Center, Beijing Institute of Genomics (BIG), Chinese Academy of Sciences / China National Center for Bioinformation (GSA: CRA002184), and are publicly accessible at https://bigd.big.ac.cn/gsa/.

## Supplementary material

Supplementary data 3

Supplementary Figure S1 ***BRAF* V600E mutation and BRAFi resistance of M238R1**. **A.** Growth curves for the parental melanoma cell line M238 and the isogenic BRAFi-resistant sub-line M238R1. Cells were treated with PLX for 72 h. **B.** The codon encoding valine at amino acid position 600 was mutated to encode glutamic acid (highlighted in red). M238R1 cell line is BRAF^V600E^ heterozygous.

Supplementary Figure S2 **The quality control measurements of the M238R1 CRISPR screens. A.** Read counts and mapping ratio for both replicates (A, B). Day 0 samples are harvested from the M238R1 cells before treatment after puromycin selection. For DMSO or PLX samples, M238R1 cells were further cultured for 10 × doubling time in the presence of DMSO or PLX4720, respectively. **B.** Number of missing sgRNAs. **C.** Gini index, the measurement of read evenness within samples. **D.** Violin plot of beta score for M238R1 cells under DMSO and PLX treatment, respectively.

Supplementary Figure S3 **Analysis of positively and negatively selected genes in CRISPR screens. A.** Positively and negatively selected genes in M238R1 cell line under the DMSO treatment. The red dots indicate the positively selected genes whose depletion provides a growth advantage under the experimental condition. The blue dots indicate the negatively selected genes which are essential for cell growth of M238R1 cells. **B.** Positively and negatively selected genes in M238R1 cell line under the PLX treatment. The pathway enrichment analysis of the negatively selected genes (blue dots) in M238R1 cell line treated with DMSO **(C)** and PLX **(D)**.

Supplementary Figure S4 **Comparison of beta scores of genes between M238R1 cells treated with DMSO and PLX. A.** Density plot of difference in beta scores comparing PLX treatment condition with DMSO treatment condition. Delta is used to measure the change of beta scores between the two conditions, which was calculated according to the formula shown in the plot. σ is chosen such that the (1-*p*) empirical quantile of the absolute values of β matches the (1-*p*/2) theoretical quantile of the prior normal distribution N (0, σ^2). *p* is set as 0.32 for 1 standard deviation and 0.05 for 2 standard deviations, which corresponds to 68% and 95% of the data falling within 1 and 2 standard deviations away of the mean, respectively. Here the delta score of our screen data is calculated to be 0.134. If the differential beta score of a gene is higher than delta (the red line), essentiality of this gene decreases after PLX treatment. If the differential beta score of a gene is lower than minus delta (the blue line), essentiality of this gene increases after PLX treatment. **B.** The beta score of M238R1 cell line under the treatment of. The two diagonal lines indicate +/–1 of the difference delta value of beta scores between DMSO and PLX treatments. The horizontal red line indicates the cutoff beta score (-0.279) of the essential genes for cell growth of M238R1 under BRAFi treatment. Genes with increased and decreased beta score after PLX treatment were indicated with red and blue dots, respectively.

Supplementary Figures S5 **The differences in gene expression in M238 and M238R1 cells treated with DMSO and PLX. A.** Volcano plot of the differentially expressed genes between the PLX and DMSO treatments in BRAFi-sensitive cell line (M238). The blue and red dots indicate down-regulated and upregulated genes in M238 under PLX treatment in comparison with DMSO treatment. The horizontal and vertical lines indicate the cutoff values (absolute FC ≥ 1.5; adjusted *P* ≤ 0.05). **B.** Pathway enrichment analysis of the down-regulated genes (blue dots in panel A) in M238 under PLX treatment in comparison with DMSO treatment. **C.** Volcano plot of the differentially expressed genes between the PLX and DMSO treatments in BRAFi-resistant cell line (M238R1).

Supplementary Figure S6 **The dependency of cell cycle genes in M238R1 cells between different conditions. A.** Boxplot of normalized read counts of sgRNAs targeting *CDK6* in M238R1 cell line at Day 0, and after DMSO or and PLX treatment. Two‐sided Wilcoxon signed rank test was performed for significance analysis, **, *P* < 0.01; *, *P* < 0.05; NS, not significant. B. Beta scores of genes encoding CDKs in M238R1 cells under the treatment of PLX (blue dots) and DMSO (red dots).

Supplementary Figure S7 **ATAC-seq data quality control for M238 and M238R1 cells treated with DMSO and PLX. A.** The median sequence quality score. **B.** Unique mapping ratio. **C.** PBC, which is defined as the number of locations with only one read divided by the number of unique locations. **D.** FRiP score, which is defined as the fraction of mapped reads that are located in the called peaks region. **E.** The number of peaks called by MACS2 with the 10-fold change. **F.** The DHS ratio estimated ratio of reads falling in DHS regions. The red lines indicate the cutoff of good quality data. The cutoff was learned from the mass of epigenetic data by the Cistrome DB. PBC, PCR bottleneck coefficient; FRiP, fraction of non-mitochondrial reads in peak region; DHS, DNaseI hypersensitive.

Supplementary Figure S8 **The dependency and ChIP-seq profiling of ETV5. A.** ChIP-seq peak signal of JUN, JUNB, ETV5 in *CDK6* genomic region. Boxplot (**B**) and segment plot (**C**) of normalized read count of sgRNAs that target *ETV5* in M238R1 cell line at Day 0, and after DMSO or and PLX treatment. Two-sided Wilcoxon signed rank test was performed for significance analysis. **, *P* < 0.01; *, *P* < 0.05; ns, not significant.

Supplementary Figure S9 ***BRAF* V600E mutation and BRAFi resistance of M229R5. A.** Growth curves for the parental melanoma cell line M229 and the isogenic BRAFi-resistant sub-line M229R5. Cells were treated with PLX for 72 h. **B.** The codon encoding valine at amino acid position 600 was mutated to encode glutamic acid (highlighted in red). M229R5 cell line is BRAF^V600E^ homozygous.

Supplementary Figure S10 **Combination synergy assay *in vitro*.** Dose response of PLX with increasing amounts of palbociclib for M238R1 (**A**) and M229R5 (**B**) cell lines. Visualization of the calculated 3D synergy maps of M238R1 (**C**) and M229R5 (**D**) cell lines.

Supplementary Figure S11 ***CDK6* signature correlated with cancer progression in patients treated with either BRAFi alone or BRAFi plus MEKi. A.** Correlation of PFS with the *CDK6* signature of tumor samples from the pre-treatment group of patients in cohort 1 who were subsequently on BRAFi treatment therapy. **B.** Correlation of PFS with the *CDK6* signature of tumor samples from the pre-treatment group of patients in cohort 2 who were subsequently on BRAFi plus MEKi treatment therapy. **C.** Correlation of PFS with the *CDK6* signature of tumor samples from the on-treatment group of patients in cohort 2 who were on BRAFi plus MEKi treatment therapy. **D.** Survival probability based on levels of *CDK6* signature in samples from the pre-treatment group of patients treated with BRAFi alone in cohort 1. **E.** Survival probability based on levels of *CDK6* signature in samples from the pre-treatment group of patients treated with BRAFi plus MEKi patients in cohort 2.
