# Supplementary material for: CRISPR Screens Identify Essential Cell Growth Mediators in BRAF Inhibitor-resistant Melanoma
Source: Genomics Proteomics Bioinformatics. 2020 May 13;18(1):26–40. doi: 10.1016/j.gpb.2020.02.002 (PMC7393575; doi:10.1016/j.gpb.2020.02.002)
Supplement: Supplementary data 1 [file mmc1.docx]

**Table S3 Pathway enrichment analysis of the 314 essential genes whose beta score decreased upon the PLX treatment compared to DMSO treatment.**

| **Pathway** | **No. of genes** | **Gene symbol** |
| --- | --- | --- |
| Cell cycle | 43 | *CDK6*, *CDK7*, *CDKN2D*, *SYCP2*, *RUVBL2*, *DNA2*, *DYNC1H1*, *CENPI*, *H2AFX*, *MAD2L1*, *MCM2*, *MCM5*, *MYC*, *ORC1*, *GINS2*, *POLD2*, *WRAP53*, *CENPJ*, *CENPN*, *RCC2*, *PSMA2*, *PSMB1*, *PSMB3*, *PSMB8*, *PSMD11*, *SPC25*, *RAD9A*, *RANGAP1*, *CCND1*, *RFC5*, *RRM2*, *CENPK*, *ACD*, *TERT*, *TUBG1*, *XPO1*, *DSN1*, *CENPT*, *CDC45*, *NUF2*, *HIST1H4E*, *RUVBL1*, *GINS1* |
| DNA strand elongation | 8 | *DNA2*, *MCM2*, *MCM5*, *GINS2*, *POLD2*, *RFC5*, *CDC45*, *GINS1* |
| DNA replication | 25 | *DNA2*, *CENPI*, *MAD2L1*, *MCM2*, *MCM5*, *ORC1*, *GINS2*, *POLD2*, *CENPN*, *RCC2*, *PSMA2*, *PSMB1*, *PSMB3*, *PSMB8*, *PSMD11*, *SPC25*, *RANGAP1*, *RFC5*, *CENPK*, *XPO1*, *DSN1*, *CENPT*, *CDC45*, *NUF2*, *GINS1* |
| G1/S transition of mitotic cell cycle | 12 | *CDK6*, *CDKN2D*, *RCC1*, *GSPT1*, *MCM2*, *MCM5*, *MYC*, *ORC1*, *CCND1*, *TAF10*, *CDC45*, *ACVR1B* |
| ERK pathway | 8 | *EGFR*, *GNB1*, *GRB2*, *HRAS*, *MYC*, *RAF1*, *SOS1*, *SRC* |
| ErbB signaling pathway | 13 | *CRK*, *CRKL*, *EGFR*, *ERBB2*, *GRB2*, *HRAS*, *MYC*, *PIK3R2*, *PTK2*, *RAF1*, *SOS1*, *SRC*, *CAMK2B* |
| EGF/EGFR signaling pathway | 14 | *MAP3K2*, *CRK*, *CRKL*, *EGFR*, *ELK4*, *ERBB2*, *GRB2*, *PIK3R2*, *PTK2*, *PTPN11*, *RAC1*, *RAF1*, *SOS1*, *ARHGEF1* |
| Ras signaling pathway | 4 | *HRAS*, *RAC1*, *RAF1*, *RELA* |
| MAPK signaling pathway | 16 | *MAP3K2*, *CRK*, *CRKL*, *EGFR*, *ELK4*, *ERBB2*, *FGFR4*, *FLT1*, *FLT4*, *GRB2*, *HRAS*, *MYC*, *RAC1*, *RAF1*, *RELA*, *SOS1* |
| Ras protein signal transduction | 6 | *CRKL*, *GNB1*, *GRB2*, *HRAS*, *SOS1*, *SETDB1* |

*Note*: Enrichment analysis was performed using R package MAGeCKFlute.
