# Supplementary material for: CRISPR Screens Identify Essential Cell Growth Mediators in BRAF Inhibitor-resistant Melanoma
Source: Genomics Proteomics Bioinformatics. 2020 May 13;18(1):26–40. doi: 10.1016/j.gpb.2020.02.002 (PMC7393575; doi:10.1016/j.gpb.2020.02.002)
Supplement: Supplementary data 2 [file mmc2.docx]

**Table S5 Mapping ratio of ATAC-seq of M238R1 and M238 cell lines**

| **Sample** | **Total No. of reads** | **No. of mapped reads** | **No. of uniquely-mapped reads** | **Unique mapping ratio** |
| --- | --- | --- | --- | --- |
| M238 DMSO | 56,023,524 | 55,143,905 | 49,778,607 | 88.85% |
| M238 PLX | 58,196,944 | 57,336,290 | 51,930,212 | 89.23% |
| M238R1 DMSO | 50,577,722 | 49,738,951 | 44,987,553 | 88.95% |
| M238R1 PLX | 43,515,798 | 42,834,440 | 38,337,619 | 88.10% |

*Note*: Read mapping of ATAC-seq data was performed using ChiLin pipeline.
