# Supplementary material for: CRISPR Screens Identify Essential Cell Growth Mediators in BRAF Inhibitor-resistant Melanoma
Source: Genomics Proteomics Bioinformatics. 2020 May 13;18(1):26–40. doi: 10.1016/j.gpb.2020.02.002 (PMC7393575; doi:10.1016/j.gpb.2020.02.002)
Supplement: Supplementary data 3 [file mmc3.docx]

**Table S8 sgRNA sequences of *AAVS1*, *CDK6*, and *ETV5***

| Gene | sgRNA ID | sgRNA oligo | sgRNA sequence |
| --- | --- | --- | --- |
| *AAVS1* | No. 1 | *AAVS1* sgRNA1F | CACCGTGCAGCTGTCTCACCCCTC |
|  |  | *AAVS1* sgRNA1R | AAACGAGGGGTGAGACAGCTGCAC |
| *CDK6* | No. 1 | *CDK6* sgRNA1F | CACCGTTCGTACTGCTGGTCAGCG |
|  |  | *CDK6* sgRNA1R | AAACCGCTGACCAGCAGTACGAAC |
|  | No. 2 | *CDK6* sgRNA2F | CACCGATGAAGAAAGTCCAGACCT |
|  |  | *CDK6* sgRNA2R | AAACAGGTCTGGACTTTCTTCATC |
|  | No. 3 | *CDK6* sgRNA3F | CACCGACTCCCAGGAGAAGAAGAC |
|  |  | *CDK6* sgRNA3R | AAACGTCTTCTTCTCCTGGGAGTC |
|  | No. 4 | *CDK6* sgRNA4F | CACCGAGCGGCATGCCCTCCTCGC |
|  |  | *CDK6* sgRNA4R | AAACGCGAGGAGGGCATGCCGCTC |
|  | No.5 | *CDK6* sgRNA5F | CACCGATACTTCCAGGACCTGGAA |
|  |  | *CDK6* sgRNA5R | AAACTTCCAGGTCCTGGAAGTATC |
| *ETV5* | No. 1 | *ETV5* sgRNA1F | CACCGAGTCATCCTACATGAGAGG |
|  |  | *ETV5* sgRNA1R | AAACCCTCTCATGTAGGATGACTC |
|  | No. 2 | *ETV5* sgRNA2F | CACCGGACTGGAACCCGTGTGCTG |
|  |  | *ETV5* sgRNA2R | AAACCAGCACACGGGTTCCAGTCC |
|  | No. 3 | *ETV5* sgRNA3F | CACCGTCATCTTTGGCATCTGCAG |
|  |  | *ETV5* sgRNA3R | AAACCTGCAGATGCCAAAGATGAC |
