# Supplementary figures and images for: CRISPR Screens Identify Essential Cell Growth Mediators in BRAF Inhibitor-resistant Melanoma
Source: Genomics Proteomics Bioinformatics. 2020 May 13;18(1):26–40. doi: 10.1016/j.gpb.2020.02.002 (PMC7393575; doi:10.1016/j.gpb.2020.02.002)

A

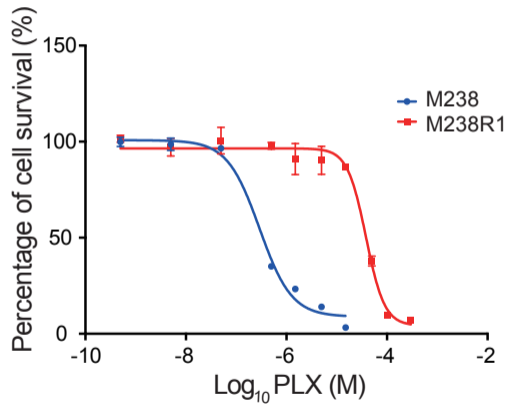

B

*BRAF* WT

M238

M238R1

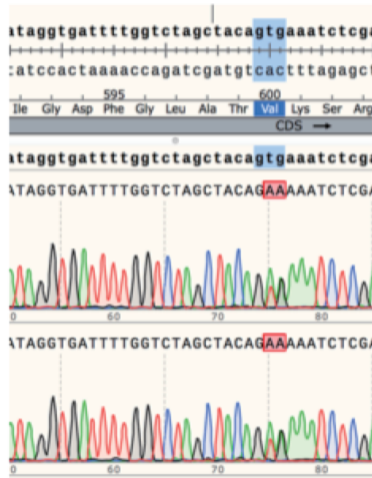

Supplement: Supplementary Figure S1 — BRAF V600E mutation and BRAFi resistance of M238R1. A. Growth curves for the parental melanoma cell line M238 and the isogenic BRAFi-resistant sub-line M238R1. Cells were treated with PLX for 72 h. B. The codon encoding valine at amino acid position 600 was mutated to encode glutamic acid (highlighted in red). M238R1 cell line is BRAFV600E heterozygous. [file mmc4.pdf]

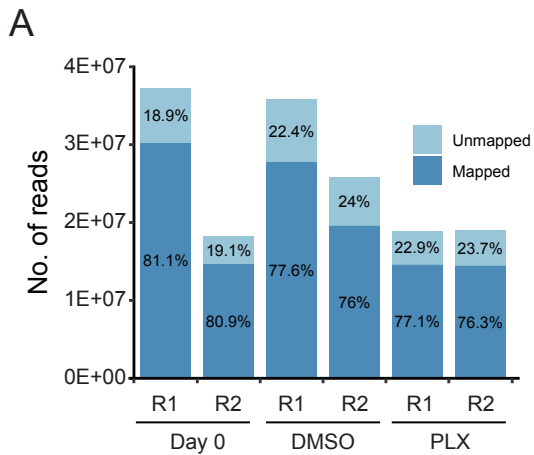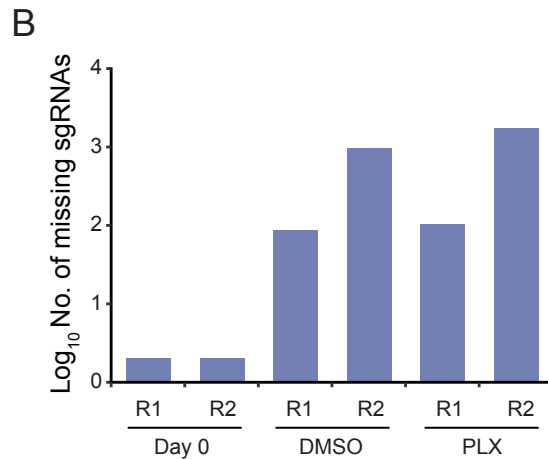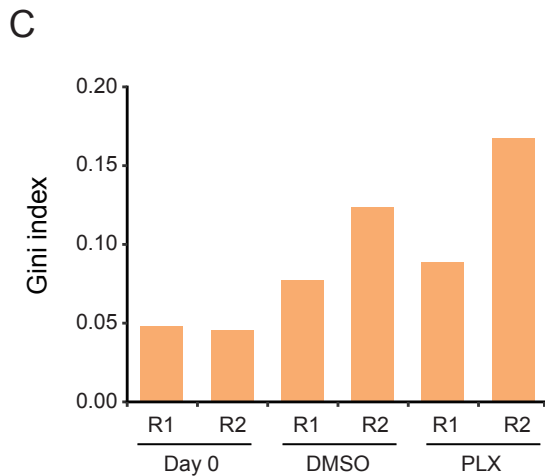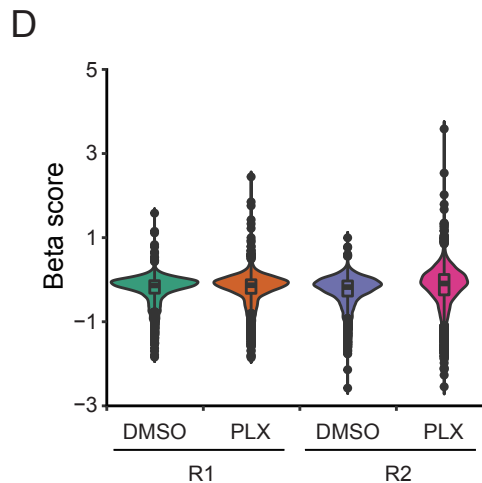

Supplement: Supplementary Figure S2 — The quality control measurements of the M238R1 CRISPR screens. A. Read counts and mapping ratio for both replicates (A, B). Day 0 samples are harvested from the M238R1 cells before treatment after puromycin selection. For DMSO or PLX samples, M238R1 cells were further cultured for 10 × doubling time in the presence of DMSO or PLX4720, respectively. B. Number of missing sgRNAs. C. Gini index, the measurement of read evenness within samples. D. Violin plot of beta score for M238R1 cells under DMSO and PLX treatment, respectively. [file mmc5.pdf]

A

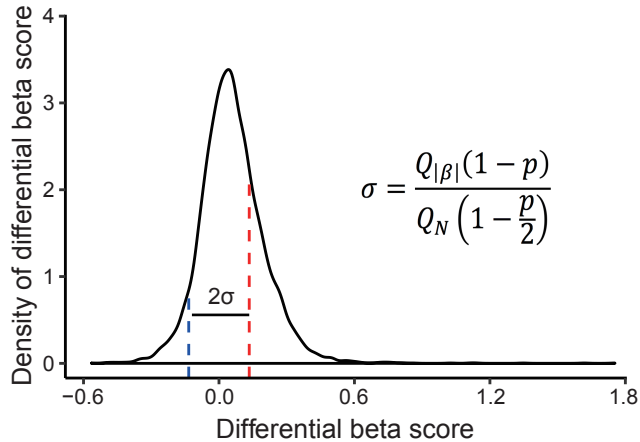

B

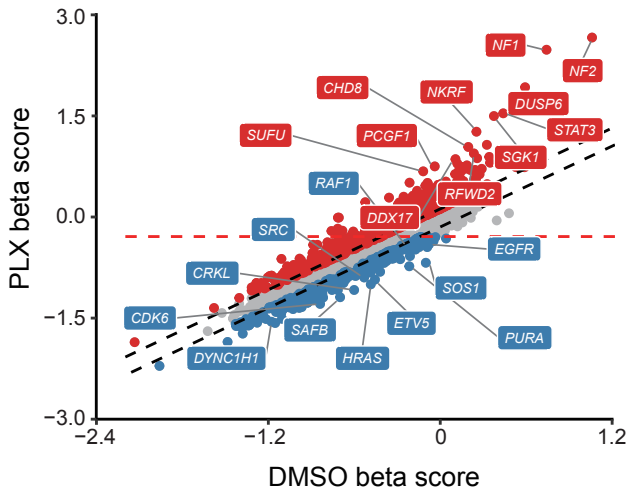

Supplement: Supplementary Figure S4 — Comparison of beta scores of genes between M238R1 cells treated with DMSO and PLX. A. Density plot of difference in beta scores comparing PLX treatment condition with DMSO treatment condition. Delta is used to measure the change of beta scores between the two conditions, which was calculated according to the formula shown in the plot. σ is chosen such that the (1-p) empirical quantile of the absolute values of β matches the (1-p/2) theoretical quantile of the prior normal distribution N (0, σ^2). p is set as 0.32 for 1 standard deviation and 0.05 for 2 standard deviations, which corresponds to 68% and 95% of the data falling within 1 and 2 standard deviations away of the mean, respectively. Here the delta score of our screen data is calculated to be 0.134. If the differential beta score of a gene is higher than delta (the red line), essentiality of this gene decreases after PLX treatment. If the differential beta score of a gene is lower than minus delta (the blue line), essentiality of this gene increases after PLX treatment. B. The beta score of M238R1 cell line under the treatment of. The two diagonal lines indicate +/–1 of the difference delta value of beta scores between DMSO and PLX treatments. The horizontal red line indicates the cutoff beta score (-0.279) of the essential genes for cell growth of M238R1 under BRAFi treatment. Genes with increased and decreased beta score after PLX treatment were indicated with red and blue dots, respectively. [file mmc7.pdf]

**A** PLX vs. DMSO  
in M238

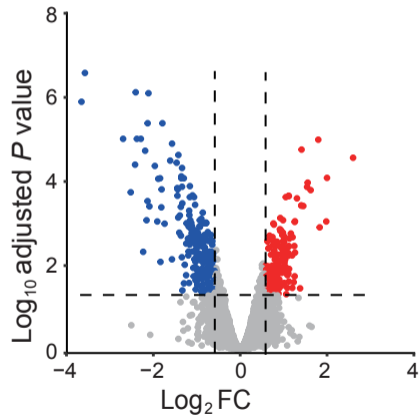

**B** Pathway analysis of down-regulated  
genes for PLX vs. DMSO in M238

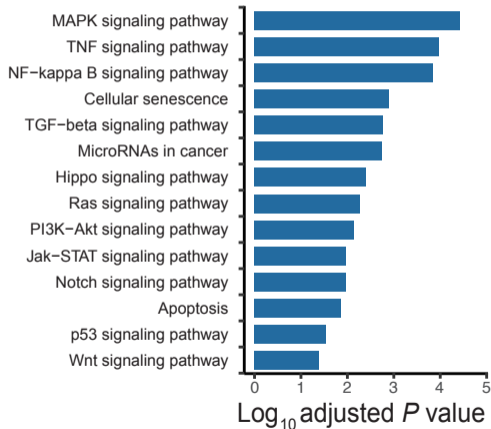

**C** PLX vs. DMSO  
in M238R1

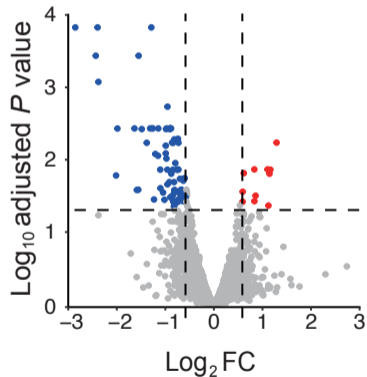

Supplement: Supplementary Figures S5 — The differences in gene expression in M238 and M238R1 cells treated with DMSO and PLX. A. Volcano plot of the differentially expressed genes between the PLX and DMSO treatments in BRAFi-sensitive cell line (M238). The blue and red dots indicate down-regulated and upregulated genes in M238 under PLX treatment in comparison with DMSO treatment. The horizontal and vertical lines indicate the cutoff values (absolute FC ≥ 1.5; adjusted P ≤ 0.05). B. Pathway enrichment analysis of the down-regulated genes (blue dots in panel A) in M238 under PLX treatment in comparison with DMSO treatment. C. Volcano plot of the differentially expressed genes between the PLX and DMSO treatments in BRAFi-resistant cell line (M238R1). [file mmc8.pdf]

A

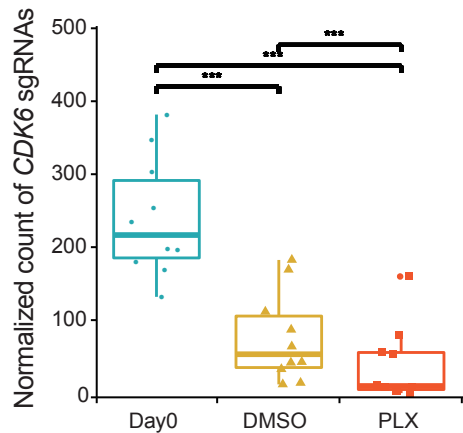

B

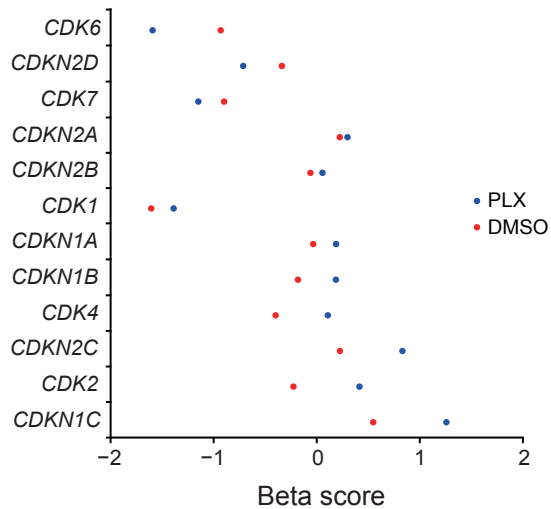

Supplement: Supplementary Figure S6 — The dependency of cell cycle genes in M238R1 cells between different conditions. A. Boxplot of normalized read counts of sgRNAs targeting CDK6 in M238R1 cell line at Day 0, and after DMSO or and PLX treatment. Two‐sided Wilcoxon signed rank test was performed for significance analysis, **, P < 0.01; *, P < 0.05; NS, not significant. B. Beta scores of genes encoding CDKs in M238R1 cells under the treatment of PLX (blue dots) and DMSO (red dots). [file mmc9.pdf]

**A**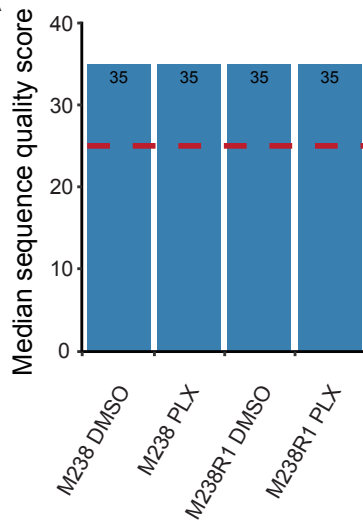**B**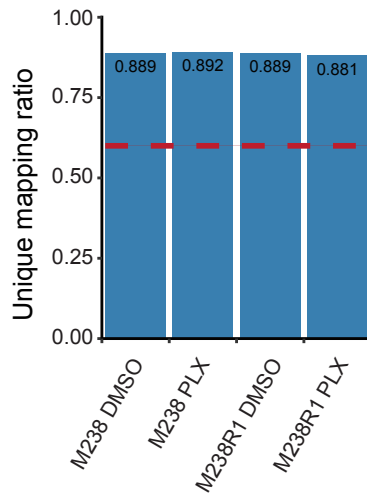**C**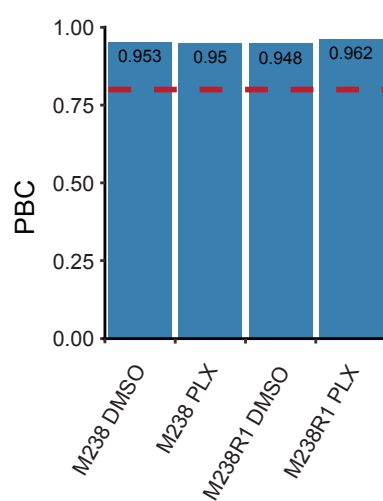**D**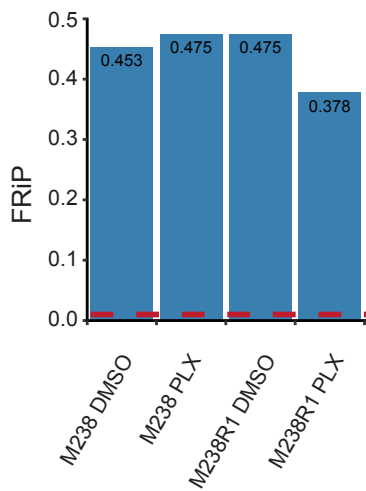**E**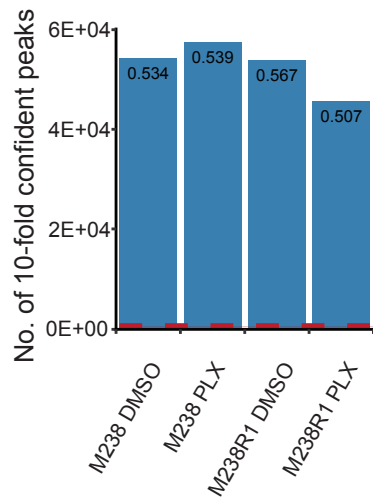**F**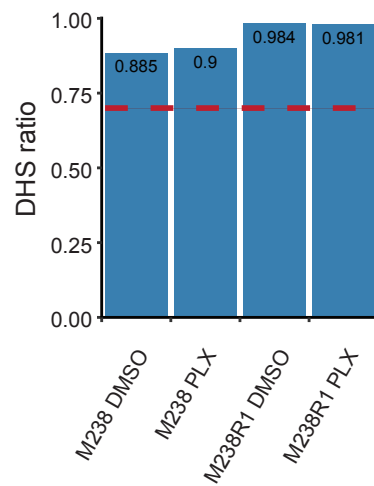

Supplement: Supplementary Figure S7 — ATAC-seq data quality control for M238 and M238R1 cells treated with DMSO and PLX. A. The median sequence quality score. B. Unique mapping ratio. C. PBC, which is defined as the number of locations with only one read divided by the number of unique locations. D. FRiP score, which is defined as the fraction of mapped reads that are located in the called peaks region. E. The number of peaks called by MACS2 with the 10-fold change. F. The DHS ratio estimated ratio of reads falling in DHS regions. The red lines indicate the cutoff of good quality data. The cutoff was learned from the mass of epigenetic data by the Cistrome DB. PBC, PCR bottleneck coefficient; FRiP, fraction of non-mitochondrial reads in peak region; DHS, DNaseI hypersensitive. [file mmc10.pdf]

A

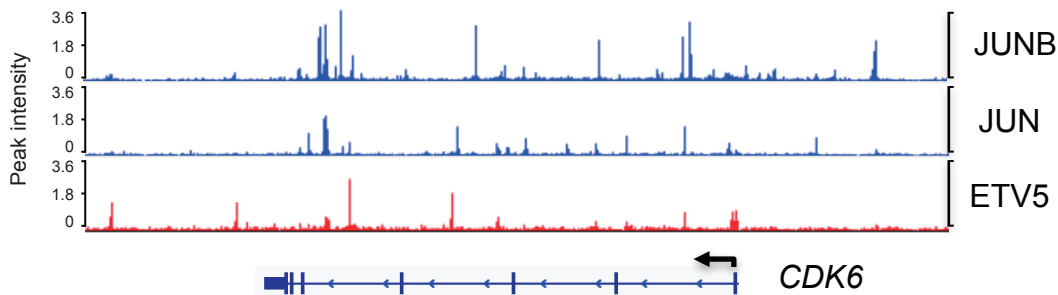

B

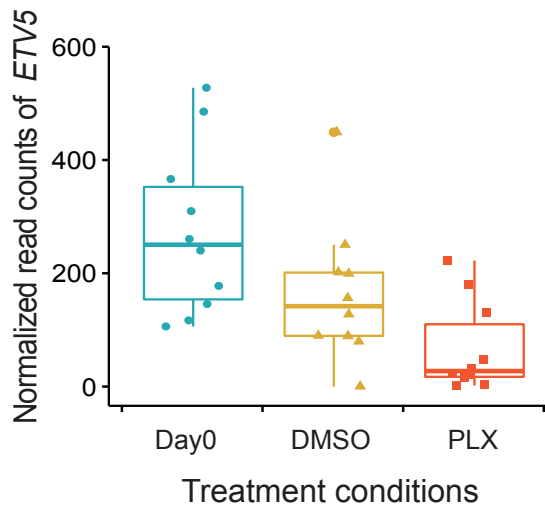

C

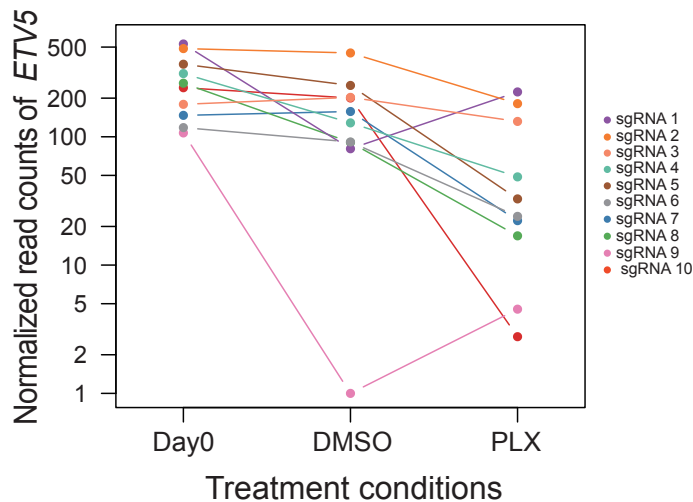

Supplement: Supplementary Figure S8 — The dependency and ChIP-seq profiling of ETV5. A. ChIP-seq peak signal of JUN, JUNB, ETV5 in CDK6 genomic region. Boxplot (B) and segment plot (C) of normalized read count of sgRNAs that target ETV5 in M238R1 cell line at Day 0, and after DMSO or and PLX treatment. Two-sided Wilcoxon signed rank test was performed for significance analysis. **, P < 0.01; *, P < 0.05; ns, not significant. [file mmc11.pdf]

A

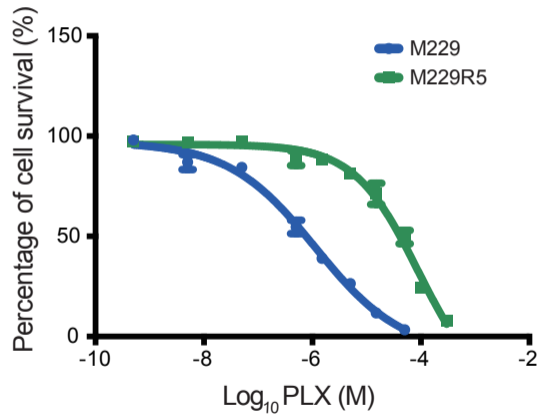

B

*BRAF* WT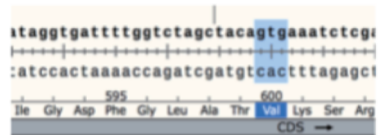

M229

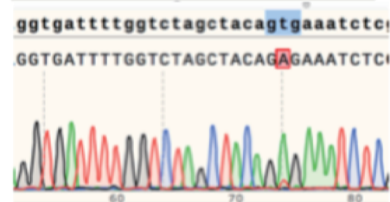

M229R5

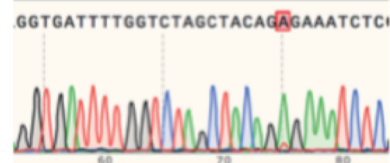

Supplement: Supplementary Figure S9 — BRAF V600E mutation and BRAFi resistance of M229R5. A. Growth curves for the parental melanoma cell line M229 and the isogenic BRAFi-resistant sub-line M229R5. Cells were treated with PLX for 72 h. B. The codon encoding valine at amino acid position 600 was mutated to encode glutamic acid (highlighted in red). M229R5 cell line is BRAFV600E homozygous. [file mmc12.pdf]

A Dose-response in M238R1

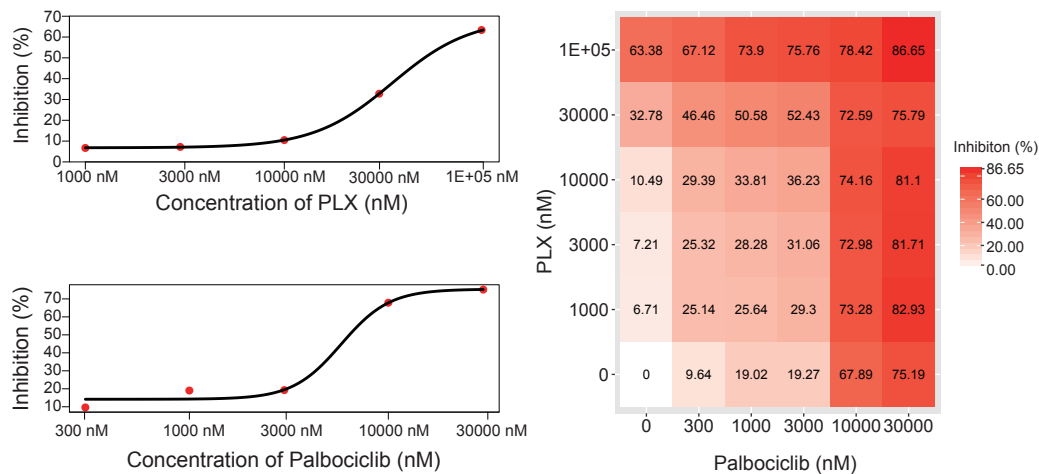

B Dose-response in M229R5

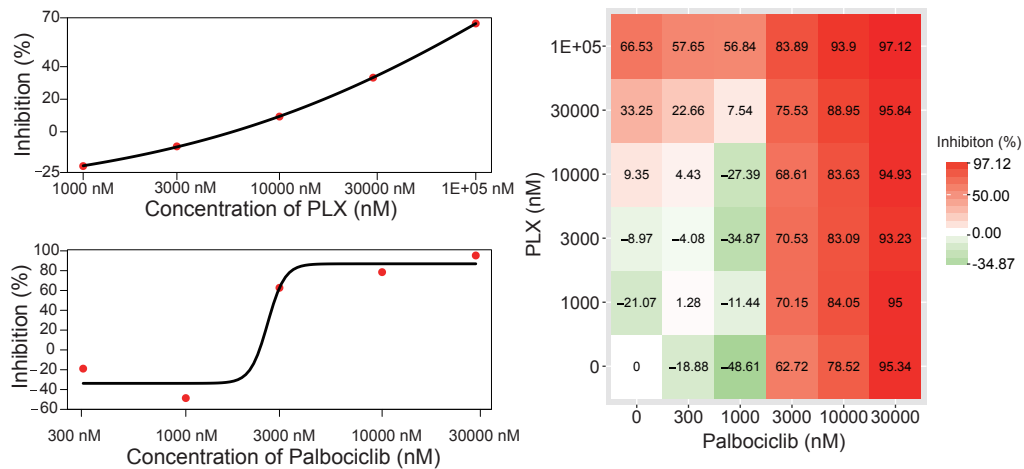

C 3D synergy map of M238R1

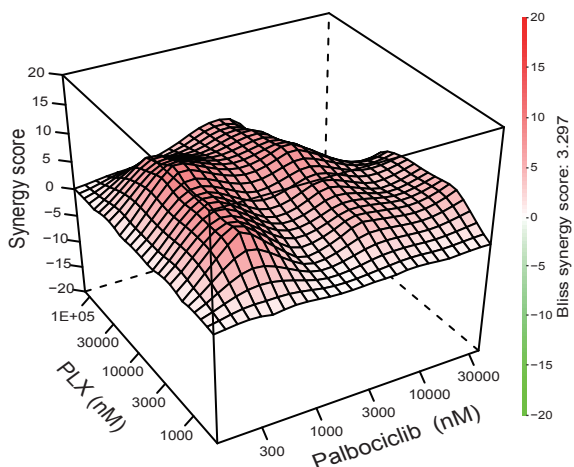

D 3D synergy map of M229R5

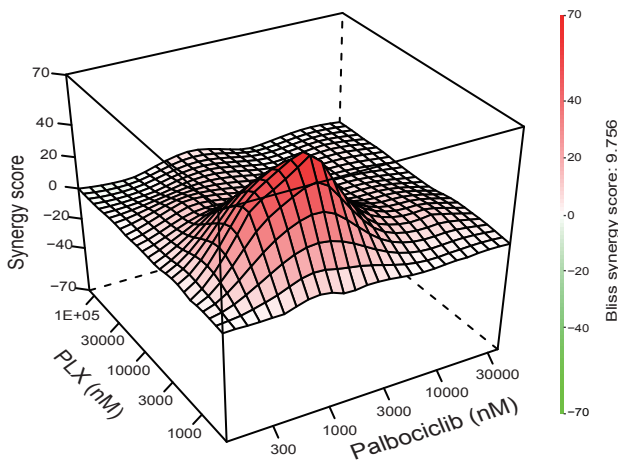

Supplement: Supplementary Figure S10 — Combination synergy assay in vitro. Dose response of PLX with increasing amounts of palbociclib for M238R1 (A) and M229R5 (B) cell lines. Visualization of the calculated 3D synergy maps of M238R1 (C) and M229R5 (D) cell lines. [file mmc13.pdf]
