# Supplementary material for: CRISPR Screens Identify Essential Cell Growth Mediators in BRAF Inhibitor-resistant Melanoma
Source: Genomics Proteomics Bioinformatics. 2020 May 13;18(1):26–40. doi: 10.1016/j.gpb.2020.02.002 (PMC7393575; doi:10.1016/j.gpb.2020.02.002)
Supplement: Supplementary Figure S3 — Analysis of positively and negatively selected genes in CRISPR screens. A. Positively and negatively selected genes in M238R1 cell line under the DMSO treatment. The red dots indicate the positively selected genes whose depletion provides a growth advantage under the experimental condition. The blue dots indicate the negatively selected genes which are essential for cell growth of M238R1 cells. B. Positively and negatively selected genes in M238R1 cell line under the PLX treatment. The pathway enrichment analysis of the negatively selected genes (blue dots) in M238R1 cell line treated with DMSO (C) and PLX (D). [file mmc6.pdf]

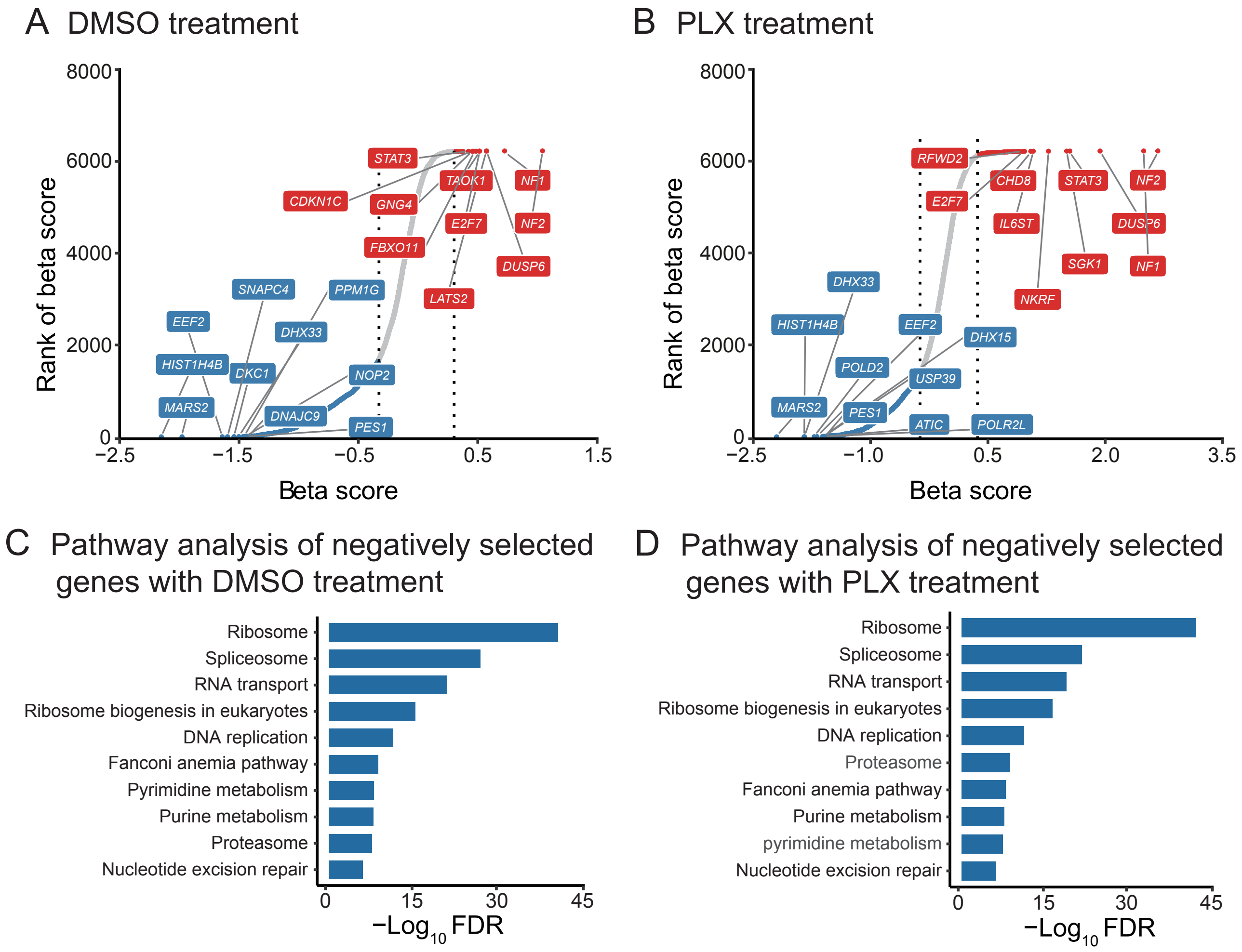

A DMSO treatment
B PLX treatment
Rank of beta score
Beta score
8000
6000
4000
2000
0
−2.5
−1.5
−0.5
0.5
1.5
−1.0
2.0
3.5
STAT3
CDKN1C
GNG4
TAOK1
NF1
E2F7
NF2
FBXO11
DUSP6
LATS2
SNAPC4
PPM1G
EEF2
DHX33
HIST1H4B
DKC1
NOP2
MARS2
DNAJC9
PES1
RFWD2
CHD8
STAT3
NF2
E2F7
IL6ST
DUSP6
SGK1
NF1
NKRF
DHX33
HIST1H4B
EEF2
DHX15
POLD2
USP39
MARS2
PES1
ATIC
POLR2L
C Pathway analysis of negatively selected genes with DMSO treatment
Ribosome
Spliceosome
RNA transport
Ribosome biogenesis in eukaryotes
DNA replication
Fanconi anemia pathway
Pyrimidine metabolism
Purine metabolism
Proteasome
Nucleotide excision repair
0
15
30
45
−Log10 FDR
D Pathway analysis of negatively selected genes with PLX treatment
Ribosome
Spliceosome
RNA transport
Ribosome biogenesis in eukaryotes
DNA replication
Proteasome
Fanconi anemia pathway
Purine metabolism
pyrimidine metabolism
Nucleotide excision repair
0
15
30
45
−Log10 FDR
