# Supplementary material for: CRISPR Screens Identify Essential Cell Growth Mediators in BRAF Inhibitor-resistant Melanoma
Source: Genomics Proteomics Bioinformatics. 2020 May 13;18(1):26–40. doi: 10.1016/j.gpb.2020.02.002 (PMC7393575; doi:10.1016/j.gpb.2020.02.002)
Supplement: Supplementary Figure S11 — CDK6 signature correlated with cancer progression in patients treated with either BRAFi alone or BRAFi plus MEKi. A. Correlation of PFS with the CDK6 signature of tumor samples from the pre-treatment group of patients in cohort 1 who were subsequently on BRAFi treatment therapy. B. Correlation of PFS with the CDK6 signature of tumor samples from the pre-treatment group of patients in cohort 2 who were subsequently on BRAFi plus MEKi treatment therapy. C. Correlation of PFS with the CDK6 signature of tumor samples from the on-treatment group of patients in cohort 2 who were on BRAFi plus MEKi treatment therapy. D. Survival probability based on levels of CDK6 signature in samples from the pre-treatment group of patients treated with BRAFi alone in cohort 1. E. Survival probability based on levels of CDK6 signature in samples from the pre-treatment group of patients treated with BRAFi plus MEKi patients in cohort 2. [file mmc14.pdf]

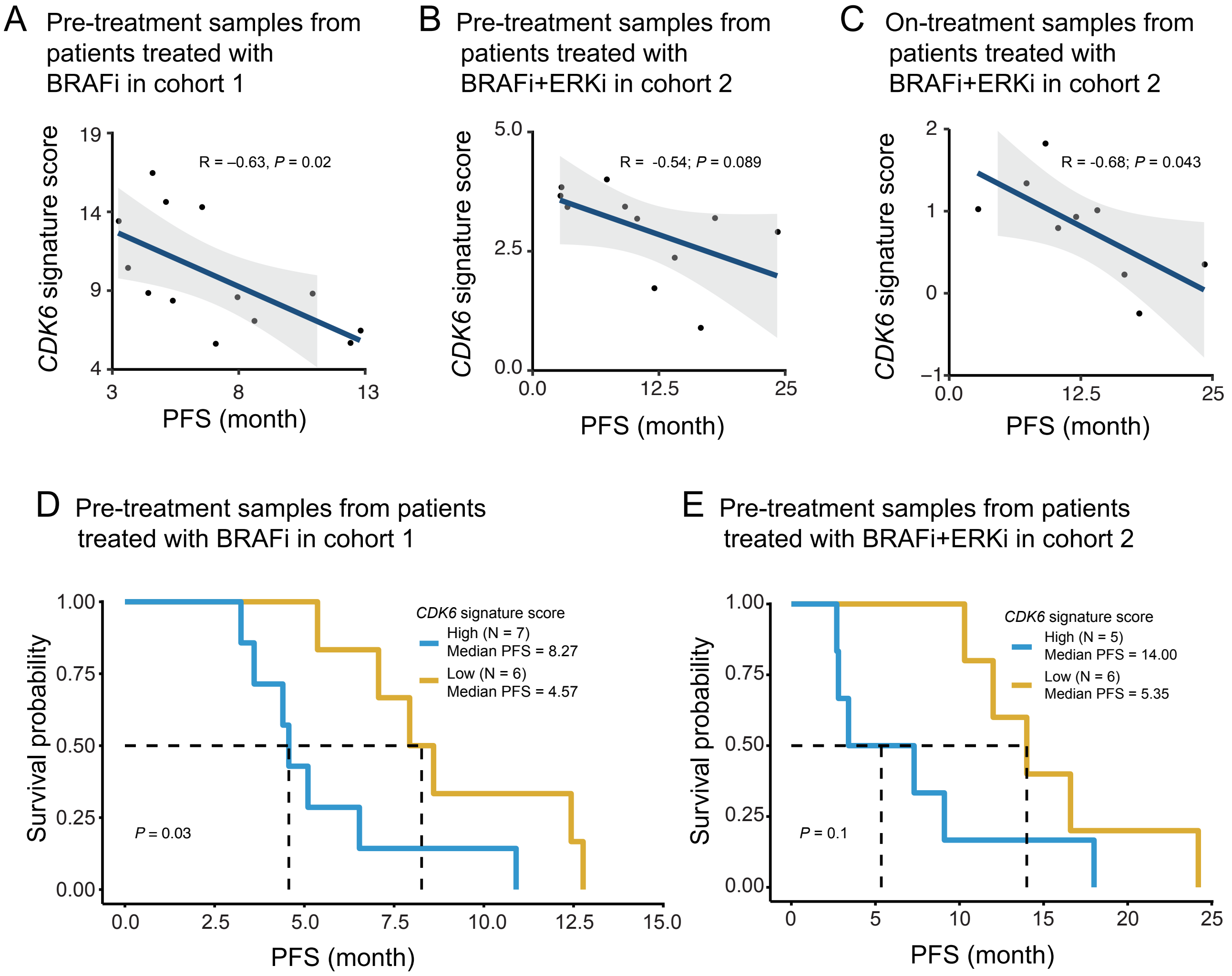
A
Pre-treatment samples from patients treated with BRAFi in cohort 1
R = –0.63, P = 0.02
CDK6 signature score
19
14
9
4
3
8
13
PFS (month)
B
Pre-treatment samples from patients treated with BRAFi+ERKi in cohort 2
R = -0.54; P = 0.089
CDK6 signature score
5.0
2.5
0.0
0.0
12.5
25
PFS (month)
C
On-treatment samples from patients treated with BRAFi+ERKi in cohort 2
R = -0.68; P = 0.043
CDK6 signature score
2
1
0
−1
0.0
12.5
25
PFS (month)
D
Pre-treatment samples from patients treated with BRAFi in cohort 1
CDK6 signature score
High (N = 7)
Median PFS = 8.27
Low (N = 6)
Median PFS = 4.57
P = 0.03
Survival probability
1.00
0.75
0.50
0.25
0.00
0.0
2.5
5.0
7.5
10.0
12.5
15.0
PFS (month)
E
Pre-treatment samples from patients treated with BRAFi+ERKi in cohort 2
CDK6 signature score
High (N = 5)
Median PFS = 14.00
Low (N = 6)
Median PFS = 5.35
P = 0.1
Survival probability
1.00
0.75
0.50
0.25
0.00
0
5
10
15
20
25
PFS (month)
